# An Auxiliary Hybrid Heuristic Approach for Objective Function Design Evaluation—Using Train Unit Scheduling as an Example

**DOI:** 10.1007/s43069-025-00529-7

**Published:** 2025-08-14

**Authors:** Li Lei, Raymond Kwan, Zhiyuan Lin

**Affiliations:** 1 School of Computing, University of Leeds, Woodhouse Lane, Leeds, LS2 9JT West Yorkshire UK; 2 Institute for Transport Studies, University of Leeds, Woodhouse Lane, Leeds, LS2 9JT West Yorkshire UK

**Keywords:** Combinatorial optimization, Hybrid heuristics, Objective function design, Objective function evaluation, Analytic hierarchy process, Train unit scheduling

## Abstract

Real-world combinatorial optimization problems are mostly NP-hard, and often only near-optimal solutions can be obtained practically. To differentiate as fine-grained as possible the near-optimal solutions is therefore desirable. Moreover, a real-world problem may have numerous possible structural properties of concern to the practitioners, too numerous to be all elicited and incorporated as optimization criteria in an objective function. In contrast with pure heuristics, we consider hybrid (meta-)heuristics that utilize an exact solver iteratively to solve a series of significantly reduced problem instances converging to near-optimal solutions within practical time. To avoid the hybrid heuristic being stranded in a “poorly differentiated” solution space, an effective objective function design plays an important role. We propose a methodology to benchmark the effectiveness of alternative objective function designs. The main metric used is the structural similarity between the solutions obtained by the hybrid heuristic and by the exact solver. Several other solution features are also distilled and aggregated in the benchmark. This methodology is explained and demonstrated on a train unit scheduling problem tested with four alternative objective functions. The results show that two of them are significantly more effective than the others in differentiating solutions of different qualities and speeding up the solution process. Moreover, some criteria not modeled explicitly could also be satisfied implicitly in the effective objective designs.

## Introduction

### Research Motivation

Real-world combinatorial optimization problems such as scheduling and routing are mostly NP-hard and have numerous possible structural properties. Thus, their objective functions are usually a trade-off among a set of optimization criteria and also a trade-off between computational efficiency and solution quality. A perfectly designed objective function taking into account all aspects often does not exist except for some idealized cases designed for theoretical interests. In practice, the ideal situation that an objective function can rank all feasible solutions precisely is hardly achieved. The real case is usually that the solutions with very similar or even the same objective function value have rather different structural properties. This phenomenon is also observed in experiments on Train Unit Scheduling Optimization (TUSO) [1–6], which is further explained in Section 4.1. Solution approaches for combinatorial optimization problems can be classified into two types: exact methods and pure/hybrid heuristic methods. Although exact methods theoretically would produce accurate solutions, they are often computationally practical only for relatively small problem instances because of their NP-hard nature. In addition, sometimes the claimed “exact optima” are only near-optimal solutions as a result of pre-set termination conditions such as an optimality gap of the branch-and-bound tree and time limit. On the other hand, heuristic methods, either pure or hybrid, normally produce “sub-optimal” solutions, and their optimality may be difficult to prove and control. Nevertheless, heuristic methods often have capability of delivering multiple near-optimal solutions in relatively short time, and the best one is claimed as the final solution.

A final solution is determined based on relations and significance (weights) among the main optimization criteria modeled in the objective function. Taking scheduling as an example, various complex structures in different schedules represent different operational plans, and it is up to the objective design to differentiate them in their quality reflected by their objective values. It is vital for an objective function to have the ability of differentiating solutions of various structures as fine-grained as possible, especially when they are near optimal. Exact solvers may not be as much influenced by the structural properties of a problem instance as hybrid heuristics do. However, hybrid heuristics are used for real-world problems, when in practice the instances are often too large and complex for exact solvers. Hence, this paper is also motivated by the quest for good trajectories of the hybrid heuristic process in terms of structural properties in the intermediate working solutions, which may be achieved through the proposed objective function design methodology.

In this paper, we propose a systematical approach to benchmark the effectiveness of alternative objective function designs for a real-world combinatorial optimization problem that satisfies the following features:

(i) The most important criteria are not in conflict but have a discernible importance hierarchy reflected in a weighted sum objective function.
(ii) Due to the complexity of the problem, some hidden and minor criteria have to be implicitly satisfied, i.e., not directly included in the objective.
(iii) It is suitable for using hybrid heuristics, i.e., a feasible solution is formed based on a set of *components* from which only a small fraction will be used. Thus, it is possible to create various reduced instances by activating only a small subset of the components [7, 8].
(iv) There is a ready-to-use exact solver for the considered problem, and we trust that the solution obtained by the exact solver is the best as a solid benchmark. A caveat is that the exact solver alone may only be practical for small problem instances. However, it can be employed by hybrid heuristics for larger instances.

In addition, due to the complexity of the problem, we assume that the overall relations among the entire hierarchical criteria are complicated. One might either synergize or be in conflict with another, or there may be some more complicated relations unrevealed either by the model or by the practitioners. Thus, which and what optimization criteria should be considered in the weighted-sum objective is unclear at the designing stage, and even experiences from practitioners would not give a definitive answer. In this paper, we establish the concept of *effectiveness* of alternative objective function design and use this concept to measure the qualities of such designs incorporating different combinations of criteria and finally select the most desirable design to be used for large-scale instances that are beyond the computational capability of an exact solver.

### Methodology and Contributions

We use the term *auxiliary heuristics* to refer to the heuristic part in a hybrid heuristic framework, compared to the *exact* part such as an integer linear programming (ILP) solver. We propose a method using hybrid heuristics to differentiate and promote desirable structural properties in solutions and to rank alternative objective functions based on their effectiveness. This method consists of three stages:

(i) Design alternative objective functions: The main optimization criteria potentially to be included into an objective function are obtained through discussions with practitioners.
(ii) Obtain solutions and their features: An exact ILP solver (*P*) taking the full problem instance as its input is applied and used to deliver an “optimal” solution as a benchmark. An auxiliary hybrid heuristic ($P_\text {HH}$) is developed to iteratively call *P* with a significantly reduced problem instance as its input that is updated in iterations until the hybrid heuristics converge to a final sub-optimal solution. Through comparisons between the benchmark and heuristic solutions, a collection of feature values reflecting the objective function effectiveness is extracted.
(iii) Evaluate the effectiveness of alternative objective function designs: The main measure of effectiveness is based on structural similarity between various solutions found by the hybrid heuristic and the benchmark solution found by the exact solver. In conjunction with other solution features, the effectiveness of the objective function is quantified as an aggregation of solution features derived at the second stage based on analytic hierarchy process (AHP) [9].

This methodology is explained and demonstrated on the TUSO problem for which four alternative objective functions are designed and evaluated. The results show that the objective functions of higher effectiveness are able to help $P_\text {HH}$ obtain solutions with desirable differentiation properties and high structural similarities with the benchmark solution from *P*. On the other hand, the objective functions of lower effectiveness lead $P_\text {HH}$ to wander over poorly differentiated solutions whose structures are very dissimilar from the benchmark solution. For the low effective objective designs, even when the objective value of the final solution obtained by $P_\text {HH}$ is close to or identical to the benchmark from *P*, their structures are often still significantly different. Through the effectiveness analysis among different combinations of optimization criteria, the criterion that promotes/reduces the effectiveness of the objective function can also be identified and thus can be included or discarded accordingly.

While the research on multi-criteria optimization and parameter control are extensive and fruitful in literature, they mainly focus on the performance of solution algorithms. Research dedicated to establishing high confidence in objective function design and selecting desirable objective functions with respect to several complex candidate criteria with complex relations is almost blank in literature. The methodology proposed in this research bridges these two gaps to quantify the objective function effectiveness and to select a desirable candidate objective based on effectiveness. For a real-world combinatorial optimization problem such as scheduling, an exact method either produces a solution or no solution at all. Originally, to assess which objective design is more desirable, one would need to rely on the results of an exact solver (by trying all alternatives) and to also seek from experts’ domain knowledge. When the problem scale is large, an exact solver may fail in giving an optimal solution. Different experts may have different conclusions, and their judgment may be affected by subjective reasons. This newly proposed method provides a more objective and standardized way to systematically establish confidence in and evaluate alternative objective functions based on the performance and structural properties from running reduced problem instances using hybrid heuristics, thus avoiding sole reliance on an exact solver or experts.

### Organization of the Paper

This paper is organized as follows: Section 2 reviews relevant literature. Section 3 introduces the methodologies used for designing and evaluating objective function design based on hybrid heuristics. Section 4 introduces the Train Unit Scheduling Optimization (TUSO) problem and reports the computational experiment results from real-world instances of TUSO. Finally, Section 5 discusses conclusions and future work.

## Literature Review

To solve a real-world problem by optimization, model simplification is usually applied to reduce the complexity of the problem to make it easier to solve. For instance, transforming nonlinear expressions to linear ones, ignoring insignificant factors, and converting time-related parameters to static parameters. Many real-world problems have multiple optimization criteria that can be modeled as a set of mathematical objective terms $f_1(x), f_2(x), \dots , f_n(x)$, to be considered in the objective function, where $x \in X \subset \mathbb {R}^d$ are the decision variables in a *d*-dimensional space. These optimization criteria can often be conflicting, and their priorities are not easy to determine such that these problems are usually regarded as multi-criteria optimization [10]. Studies in multi-criteria optimization are extensive in literature. “Errors” or “uncertainties” arise when simplification and approximations are applied to models and solution approaches. Reduction of uncertainties is thus needed during model calibration processes such as parameter tuning and control. This section briefly reviews the literature on multi-criteria optimization and parameter control. The focus of this research is on the objective function effectiveness, which is based on an established mathematical model that has been properly simplified and a readily available exact solver whose parameters are fixed.

### Multi-Criteria Optimization

Usually, there is no definite ranking on the solutions of a multi-criteria optimization problem, i.e., no single solution can simultaneously optimize and prioritize every criterion. This is because multiple criteria are often in conflict and do not have an optimization hierarchy. Consequently, multi-criteria problems usually do not consider a unique optimal solution but a set of representative trade-off solutions. A classical methodology is to use *a priori* methods [11]. Two well-known *a priori* methods are the $\varepsilon$-constraint method and weighted sum method. The $\varepsilon$-constraint method optimizes one of the objectives and considers the others as constraints with estimated cost bounds [12]. The weighted sum method adds all terms together to be considered as a single linear weighted objective Eq. 1 by introducing weights $\alpha _i$ for each objective criterion *i*. Often, each $f_i$ is normalized by estimated bounds on its possible ranges, and the weights are restricted by Condition Eq. 2 [13].

1 $$\begin{aligned} \min _{x\in X} \quad \sum _{i=1}^{n}\alpha _i f_i(x) \end{aligned}$$

2 $$\begin{aligned} \sum _{i=1}^n \alpha _i = 1 \quad ( 0<\alpha _i <1) \end{aligned}$$

The single objective function is easier and more deterministic because it uses a unified numerical value to measure the solution quality. Unfortunately, the estimated bounds and scaled weights are usually hard to define and calibrate. The solution generated based on such given bounds and weights may not be preferred by users. On the other hand, the Pareto front technique aims to compute a set of dominant trade-off solutions for users to choose [14]. However, the number of solutions required to accurately represent the Pareto front increases exponentially as the number of objectives goes higher. The storage and time requirements of related indicators, such as hypervolume, diversity measure, and hyperarea difference, also increase exponentially with the number of objectives [15].

Many train planning and scheduling problems can be considered as multi-criteria optimization problems. For instance, a multi-objective model is proposed to deal with the passenger train scheduling problem [16]. Two conflicting criteria are considered: railway company-view optimization criteria and passenger-view optimization criteria such that fuel consumption and traveling time have to be both minimized. In [17], a two-objective integer programming model is proposed to describe the train utilization and operation problem in a subway system, which is solved by a genetic algorithm. In [18], a multi-objective formulation is presented to assess mainline train services considering the interests of multiple stakeholders, such as journey times, customer waiting times, punctuality, and crowdedness. Similarly, a genetic algorithm is applied. The trade-off between conflicting objectives is illustrated through the Pareto analysis.

Multi-criteria optimization models in the literature mainly concern Pareto fronts and their analysis, in which conflicting multi-objectives have individual objective functions that are not merged. However, the multiple objectives considered in this research are not in strong conflict and have a discernible importance hierarchy that can be merged into a single weighted sum objective function.

### Parameter Tuning and Control

Parameter tuning and control are usually studied after objective functions are determined and solution approaches are developed. It tunes the parameters affecting the time needed for an algorithm to find a better solution but does not contribute to the “definition” of what is considered as optimal. Conventionally, most mathematical algorithms have default parameter settings that are manually set in an ad-hoc manner via considerable efforts on experiments and from experiences [19]. For instance, there are 80 parameters in CPLEX affecting the search mechanism that can be controlled by users [20]. In the last few decades, many automatic methods for parameter tuning and control are reported in literature, particularly in (meta-)heuristic methods. They are classified into four types [21]: (i) sampling methods, (ii) model-based methods, (iii) screening methods, and (iv) meta-evolutionary methods. Sampling methods reduce search effort by cutting down the number of parameters. However, this leads to the challenge of predicting a limited number of parameters that have the best performance and robustness. A sampling method to systematically tune parameters (up to five) is proposed in [22], which is based on statistical analysis and local search techniques. The second type of method establishes models based on parameter data to reduce the total number of experiments. For instance, the sequential model-based algorithm configuration (SMAC) method based on random forests is model-based [23], which expands this method to general algorithm configuration problems. Screening methods identify the best parameters with a minimum number of experiments. For example, “*-Race” approaches are commonly used [24, 25]. Meta-evolutionary methods consider parameter tuning as an optimization problem, for instance, the parameter iterated local search (ParamILS) method [20] and the focused iterated local search (FocusedILS) method [26], which have inspired many other studies on configurations of multi-objective problem [27].

However, few researches discuss weight tuning for objective function terms. In [28], AHP is used to assign the weights of penalized terms in the objective function acting as soft constraints of a nurse scheduling problem. Based on historical data, a method to automatically determine the relative importance of soft constraints is proposed in [29]. In the area of train planning and scheduling, weights of objective function terms are mostly set as pre-determined parameters, and the method of deriving those weights is not explained in detail [2, 30, 31].

In this research, the main focus is to evaluate the effectiveness of alternative objective function designs formed as different combinations of the main optimization terms. The weight of each term is set based on the hierarchical importance based on experiences and experts’ domain knowledge.

### Hybrid Heuristics Combining Exact Solver with Reduced Instances

Hybrid heuristics (HH), or sometimes referred to as hybrid meta-heuristics [32, 33], are a class of optimization methods particularly designed for solving difficult combinatorial optimization problems. In principle, any optimization algorithm combining heuristics with at least one other optimization approach can be regarded as “hybrid heuristics.” [32] and [7] give in-depth surveys on the definition, taxonomy, and relevant work in hybrid heuristics for solving combinatorial optimization problems.

In this paper, we only focus on a type of hybrid heuristics that uses the following strategy: An exact solver (usually an ILP solver) is available, but it may only be able to solve small to medium-sized instances. An auxiliary heuristic is thus designed to iteratively reduce the problem instance into a suitable size that can be solved quickly by the exact solver. The quality of an initial solution based on the reduced instance will often be low, but customized strategies are designed to update the reduced instances so that, in the final rounds, the reduced instance will be expected to contain most components needed for producing an optimal or near-optimal solution. We give some examples of the above restrictively defined HH, which we found are closely relevant to the HH to be used in our alternative objective function design approach. They are not meant to be exhaustive, and more comprehensive reviews on other examples can be found in [33] and the survey paper [7].

In [34], a hybrid heuristics named PowerSolver is proposed to solve large and/or complex driver scheduling problems [35]. Column generation is used in solving bus and train driver scheduling problems where each column corresponds to a potential driver shift. The number of columns in the set covering ILP model could run into billions, making the problem intractable. PowerSolver derives a series of small refined sub-problem instances fed into an existing efficient set covering ILP-based solver. Instances are reduced by banning the use of most relief opportunities (RO, where/when a driver change can take place). In each iteration, a minimum collection of RO is retained such that the next solution will be no worse than the current best. Controlled by a customized user strategy, a small number of the banned relief opportunities would be reactivated, and some soft constraints may be relaxed before the new sub-problem instance is solved. PowerSolver gives a step-change in fully automating UK train operating companies’ crew scheduling in large/complex real-world scenarios. It is proved successful by many transport operators who are routinely using it as a key component of TrainTRACS, a commercial crew scheduling software package [36].

Like in crew scheduling, challenges in solving large scale instances also occur in the TUSO problem. In [37], an HH approach named size limited iterative method (SLIM) is proposed, where arcs in a DAG represent train unit connection opportunities. Since the exact solver (RS-Opt) [2] is only able to solve small to medium-sized instances in a reasonable time, SLIM will reduce problem instances by removing the majority of the arcs in the DAG while still ensuring the existance of feasible solutions and the quality of the series of solutions based on reduced instances will be improving until convergence. By implementing SLIM, the train unit scheduling system can successfully solve large-scaled instances with high-quality solutions.

Both PowerSolver and SLIM follow the paradigm that we generalize as “Extract-and-Augment” in Section 3.2. In each iteration, an essence (“backbone”) of the previous iteration or iterations will be retained to ensure the feasibility and quality of the next solution. Diversification is realized by augmenting the backbone by strategically adding components from different “corners” of the solution space or “wheel rotation.” A balance between local optimality (convergence) and global optimality (diversity) can thus be achieved by tuning the strategies in extracting the backbone and augmentation by wheel rotations.

Reference [8] presents a general hybrid metaheuristic for combinatorial optimization named Construct, Merge, Solve & Adapt (CMSA). It is a specific instantiation of a framework known as “generate-and-solve” [38]. CMSA generates a reduced sub-instance of the original problem, which a solution to the sub-instance is also a solution to the original problem. An exact solver is applied to the reduced sub-instance to obtain a high-quality solution to the original problem instance. A mechanism is developed to make use of the results of the exact solver as feedback for the next algorithm iteration. Two examples are tested to prove the effectiveness of CMSA: the minimum common string partition problem and the minimum covering arborescence problem. The obtained results show that CMSA is competitive with the exact solver for small to medium-sized problems, and it significantly outperforms the exact solver for larger-sized problems.

More recently, CMSA has been successfully applied in several instances and compared with other heuristics. In [39], it is used to solve an NP-hard problem from the family of dominating set problems in undirected graphs, where the minimum positive influence dominating set problem is studied. The experiments show that CMSA outperforms the current state-of-the-art metaheuristics from the literature. Moreover, CMSA is able to find competitive solutions as found by CPLEX for small and medium-sized instances and clearly outperforms CPLEX for large instances. In [40], a comparative analysis is given on two hybrid algorithms for combinatorial optimization problems, i.e., large neighborhood search (LNS) and CMSA. The multidimensional knapsack problem and minimum common string partition are used for the comparisons in the experiments. The results show the advantage of CMSA over LNS for instances with fewer items. LNS may perform better if there are many items. A new way of visualizing the trajectories of the compared algorithms in terms of merged monotonic local optima networks also supports the conclusions. In [41], CMSA is applied to the Prioritized Pairwise Test Data Generation Problem where a minimal subset of product family needs to be found to test all these possible valid feature combinations in software product lines. The problem can be formulated as an integer linear program. The computational experiments show that CMSA is statistically significantly better in terms of quality of solutions in most of the instances compared to other algorithms, although it requires more execution time. CMSA also shows advantages in many other problems such as the repetition-free longest common subsequence problem [42] and the nuclear power plant refueling and maintenance planning [43].

CMSA and Extract-and-Augment share many common features and design philosophy, especially in the ways they use an exact solver to find out high-quality components by solving reduced instances iteratively and the need to update an instance in each iteration based on historic feedback. They mainly differ in how a reduced instance is constructed and how diversification is realized. In CMSA, they are achieved by merging a collection of diversified feasible solutions (known as the construction phase). In Extract-and-Augment, they are done by the augmentation phase through wheel rotation by strategically considering different sectors of the problem space.

## Methodology for Evaluating Objective Effectiveness

This section describes a methodology for evaluating the effectiveness of alternative objective function designs. The development of this methodology was inspired by experimental investigations on the TUSO problem aforementioned in Section 1.1. TUSO is modeled as an integer multi-commodity flow problem on a pre-generated directed acyclic graph (DAG). A feasible solution only uses a small fraction (subgraph) of the full DAG where commodity flows represent train units serving train trips represented by nodes. Therefore, the size of the full DAG directly affects the computational complexity of solving the network flow problem because of enormous arc-and-flow combinations. An exact solver called RS-Opt has been developed for small/medium instances [2]. A hybrid heuristic method named SLIM has also been developed for larger instances [37]. Without SLIM, RS-Opt takes the entire DAG as input and delivers an “optimal” solution $s^*$. SLIM aims at iteratively extracting, reducing and refining the full DAG into a small subgraph to feed to RS-Opt such that RS-Opt can yield a solution quickly. At the convergence of SLIM, a solution $\hat{s}$ with an objective value close to $s^*$ is expected, i.e., $|z(s^*)-z(\hat{s})|\le \varepsilon _1$ measured by some tolerance $\varepsilon _1$. More importantly, it is also expected that $\hat{s}$ should possess similar *structural properties* compared with $s^*$. In other words, if we define a metric $sim(\cdot )$ for measuring structural similarities on solutions, ideally it should also be the case that $|sim(s^*)-sim(\hat{s})|\le \varepsilon _2$ with some tolerance $\varepsilon _2$. However, it is often observed that the structural properties of $s^*$ and $\hat{s}$ are very different, although their objective values are close or even the same. Although the branch-and-price process inside RS-Opt may have its own influence on the arc (component) selection over the DAG, certain deficiencies in the design of objective function being not sensitive enough to differentiate solution structures may have also contributed to the unexpected phenomenon. In addition, it is observed that some objective combinations lead to quick convergence, while some others show very slow progress. Interestingly, there is often a strong correlation between structural similarities at optima and convergence speed: the more similar, the quicker.

All these show a need to create a systematic approach to design and evaluate the effectiveness of alternative objective functions. We measure the effectiveness by a multiple of solution features including (i) a unified solution quality score (regardless of specific objectives in use), (ii) structural similarities, (iii) number of different solutions, and (iv) comparison between the objective values of the HH method and the exact solver. The details of these features will be given Section 3.3.

**Fig. 1:**
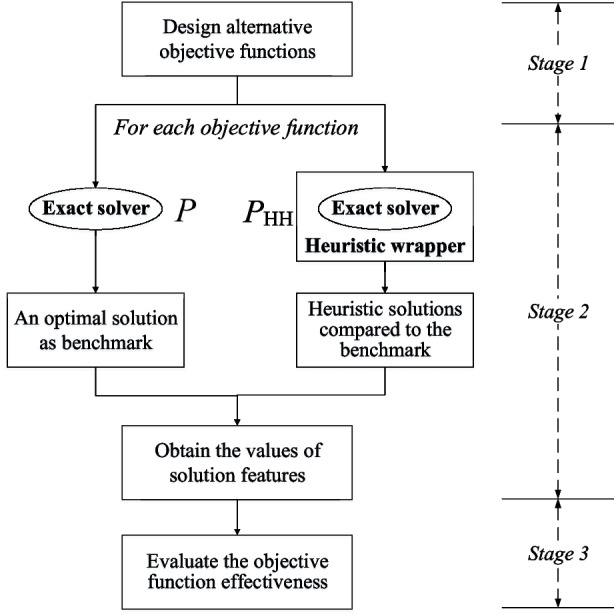
Methodology flowchart

Figure 1 gives a flowchart on the concept of this approach for evaluating objective effectiveness, where three stages are involved. The first stage is to design several alternative objective functions where each may have its own pros and cons from the designer’s point of view. A set of solution features is also created and collated based on the objective design and the assumption that an exact solver *P* and a HH framework $P_{\text {HH}}$ are available. There can be multiple layers of features. For instance, the first layer features are $\Phi _1,\Phi _2,\dots ,\Phi _m$, and within each first layer, second layer features can be further defined as $\phi _{11},\phi _{12},\dots ,\phi _{21},\phi _{22},\dots ,\phi _{m1},\phi _{m2},\dots$. In the second stage, for each objective function design, this method uses the exact solver *P* and the hybrid heuristic $P_\text {HH}$ that iteratively creates reduced instances to obtain a series of solutions. The solution from *P* is considered as a benchmark, and the heuristic solutions obtained from $P_\text {HH}$ are compared with the benchmark. Through the comparisons, a collection of feature values $v_{11},v_{12},\dots ,v_{21},v_{22},\dots ,v_{m1},v_{m2},\dots$ corresponding to the solution features $\phi _{ij}$ is obtained and will be used as the raw data for Stage 3.

In the third stage, the effectiveness of each objective function design is quantified by hierarchically integrating all the solution feature values $$v_{11},v_{12},\dots ,v_{21},v_{22},\dots ,v_{m1},$$$$v_{m2},\dots $$ into an overall metric $$M(\cdot )$$. For instance, AHP is a classical method for such an integration where an adjusted final weight $$\hat{w}_{ij}$$ for each second layer $$\phi _{ij}$$ is calculated. The overall metric is thus calculated as a function of $$v_{ij}$$ and $$\hat{w}_{ij}$$, e.g., $$M(\hat{w},v) = \sum _{ij} \hat{w}_{ij} v_{ij}$$, if a simple weighted linear combination is used. To get reliable and realistic values on feature weights, practitioners are often involved at this stage to review if the evaluation results are in line with their judgement. We elaborate each stage in the remaining of Section 3.

### Alternative Objective Function Designs

Stage 1 aims at creating several candidate objective functions through problem investigation. The participation of practitioners is important at this stage, especially in identifying main optimization criteria with discernible importance. The selected criteria are only a subset from a theoretically complete list of criteria for the considered problem. The optimization criteria from the perspective of practitioners are often descriptive and macroscopical. Modeling them as mathematical terms is a vital step to enable the problem to be solved by optimization methods.

As the main optimization criteria we considered in this paper respect a discernible importance hierarchy, they can be formulated in a linear weighted-sum objective function— the weights of the corresponding mathematical terms reflect a hierarchy. Denote optimization terms and their pre-determined weights as $f_1, f_2, \dots , f_n$ and $\alpha _1,\alpha _2,\dots ,\alpha _n$, where *n* is the total number of optimization terms converted from the main optimization criteria. Thus, the alternative objective functions are designed based on different combinations of these optimization terms in weighted-sum formulations. A candidate objective function indexed by *k* can be expressed as below:

3 $$\begin{aligned} F_k = \sum _{i \in I_k} \alpha _i f_i,\quad I_k \subset \{1,2,...,n\}, \end{aligned}$$

where $|I_k|<n$ terms from $f_1,\dots ,f_n$ are considered. As we have mentioned in Section 1, in reality, often *n* is rather big, and the designer can only afford a very much smaller $m \ll n$ terms to be included in the objective. The most desirable objective design is the one with only *m* items but is still able to achieve the effectiveness of the presence of most of the *n* original terms as much as possible. A set of alternative objective functions with different objective terms $I_k$ will be generated and put into a candidate set $\mathcal {F}=\{F_1,F_2,\dots ,F_k, \dots \}$. The ultimate target is to give a ranking of the candidates in $\mathcal {F}$ and choose the most desirable one.

### HH Framework

For a combinatorial optimization problem, let us define the instance corresponding to the complete original input by *G*. The components of a feasible solution *s* to the original problem *G* often correspond to a very small subset $\Gamma \subset G$ of the entire input. This is also the case for the optimal solution $s^*$ whose components form a very small subset $\Gamma ^* \subset G$. More importantly, any subset of *G* containing $\Gamma ^*$ will also yield the same optimal solution $s^*$. We call a subset of *G* that is a superset of $\Gamma ^*$ an *optimal subset* of *G* and denote it by $G^*$. Most of the components of the entire input *G* do not directly contribute to finding the optimal solution but merely increase the computational burden. Thus, deriving minimal good-quality reduced inputs as close as possible to a $G^*$ is an effective method to significantly improve computational efficiency or even enable tractability. The aim of a HH [7, 8, 34, 37] is to eventually find an appropriately reduced input instance $\widehat{G} \approx G^*$ enabling an exact method *P* to deliver a solution whose objective value is very close the “optimal” solution obtained by *P* based on the entire input *G*, i.e., $z[P(\widehat{G})] \approx z[P(G^*)] = z[P(G)]$, where we denote *P*(*X*) as the solution of using *P* to solve instance *X*. In most cases, it is not possible to find an optimal subset, i.e., $\widehat{G}=G^*$. An effective way of creating a properly reduced subset for *P* in HH is by *augmenting* an existing feasible solution [34, 37]. Often, the entire problem instance *G* can be partitioned into many (usually mutually exclusive) “regions” $G = G_1 \cup G_2 \cup \cdots$. Each region contains components that share certain common features. For instance, they may belong to the same group of an attribute identified in *G*. There are often several region types that can be used for partitioning *G* based on the characteristics of a problem, e.g., by time, location, staff allocation, and traction type. At an iteration of HH, a certain region type is in use. Problem instances are created by augmenting feasible solutions with components from a certain region(s). This is proved effective in increasing the potential of forming better structural properties in resultant solutions. Suppose we arrange the regions on a wheel. Augmenting a feasible solution by adding components from different regions on the wheel is called a *rotation* process.

**Algorithm 1:**
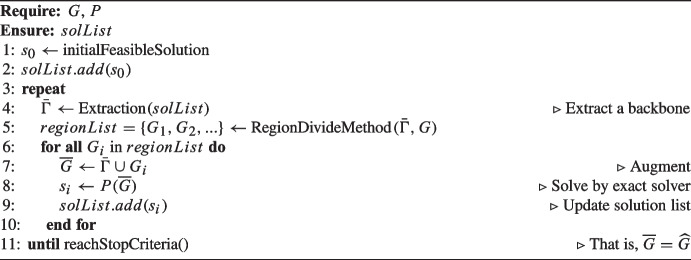
Pseudo code of $P_\text {HH}$ (Extract-and-Augment).

Thus, a heuristic approach $P_\text {HH}$ iteratively forming feasible solutions and augmenting them by region rotation to promote desirable structural properties can be used to find a large number of refined reduced instances. We denote a reduced instance by $\overline{G}$. Eventually, $P_\text {HH}$ will be terminated when there is evidence showing $\overline{G}$ is an appropriate reduced instance $\widehat{G}$ that we look for. See Algorithm 1 for a detailed description of $P_\text {HH}$, which we generalize as “Extract-and-Augment.” Applications of Extract-and-Augment, such as PowerSolver [34] and SLIM [37], are widely used in railway scheduling. Note that we present the details of Extract-and-Augment as it is the HH approach used in the experiments in Section 4. Other kinds of HH methods (such as CMSA [8]) are also potentially applicable as the heuristic part in our three-step methodology.

$P_\text {HH}$ requires a complete original input (*G*) and a ready-to-use exact solver (*P*) to carry out an iterative process. Let *solList* be a list to store all solutions found during the converging process. $P_\text {HH}$ starts with an initial feasible solution $s_0$ that can be obtained by some simple heuristics such as a greedy algorithm. The *Extraction*() method takes the *solList* as input to extract an essential subset (“backbone”) corresponding to a very small subset $\bar{\Gamma }$ of *G* to ensure the reduced input formed at this iteration has a feasible solution. *Extraction*() can be defined in many customized ways. For instance, extracting the *best* solution in *solList* could be an option, which is helpful to speed up the convergence. Randomly extracting *any* solution is also an option that has the advantage of helping $P_\text {HH}$ jump out of local optima. According to the characteristics of a specific problem, *G* can be partitioned in various ways to obtain the regions in *regionList*.

The loop on *regionList* represents the rotation on regions to ensure that every “corner” of the entire problem space is reached as much as possible. The merge between $\bar{\Gamma }$ and region $G_i$ is called *augmentation* by adding components from region $G_i$ on the “wheel.” The augmentation process results in a reduced input $\overline{G}$ to be solved by the exact solver *P*. By looping over steps of “Extract–Augment–Solve–Update,” a collection of solutions is found and stored in *solList*. The augmentation is applied systematically and, in particular, tries to divide the problem space taking care of combinatorial features for promoting good structural properties in the solutions. $P_\text {HH}$ will stop when it is believed that the best reduced instance so far $\overline{G}$ is a good approximation of the desirable $\widehat{G}$. Commonly used criteria are maximum time/iterations and/or none improvement on the objective function value for a certain number of iterations. The size of a region can be controlled through a parameter $\mu$, logically, $0<\mu \ll 1$ to make sure $|\overline{G}| \ll |G|$ such that *P* can deliver an “optimal” solution quickly [37]. On the other hand, the benchmark solution $s^*$ is obtained by considering the entire input *G* for *P*, i.e., $s^* = P(G)$. At convergence, $P_\text {HH}$ feeds an almost optimal reduced input $\widehat{G}$ to *P* to produce the final solution $\hat{s}$. Before convergence, the reduced input at each iteration is sub-optimal but is of a very small size such that *P* can be executed very quickly to claim an “optimal” solution $s_i$, which may be extracted as an essential subset (backbone) for the next iteration. A good feature of Extract-and-Augment is that given the entire input *G*, $P_\text {HH}$ can find a set of solutions $S=\{s_1,s_2,\dots , s_i, \dots , \hat{s}\}$ during the process of skimming *G* into $\widehat{G}$.

**Fig. 2:**
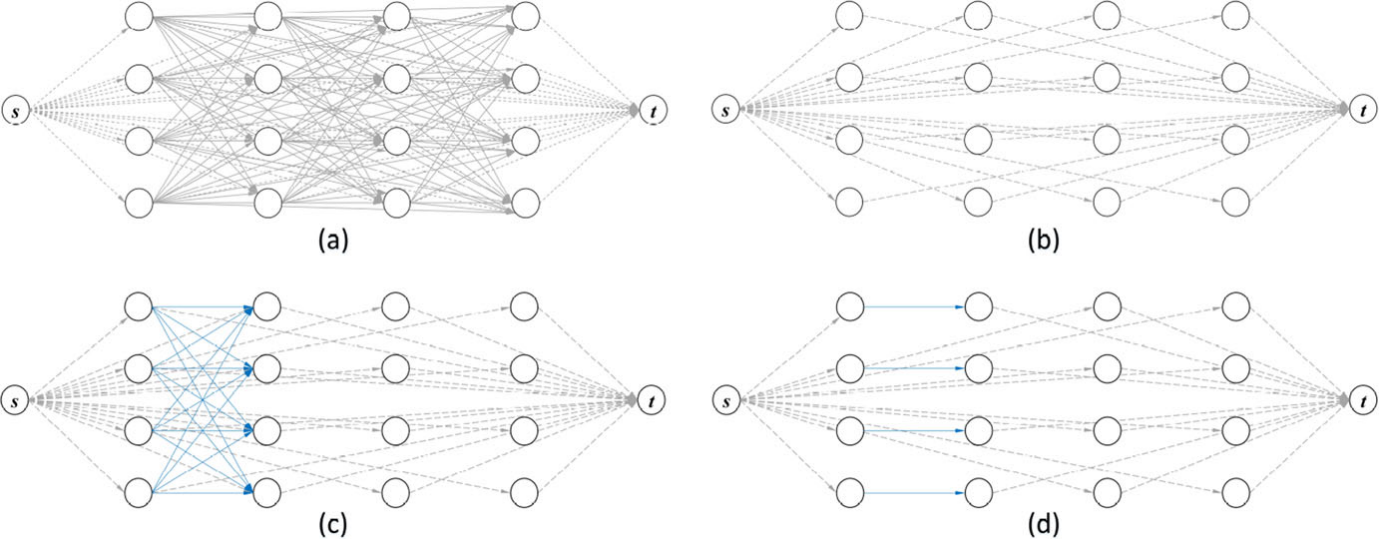
Working mechanism of $P_\text {HH}$

Consider the network flow-based TUSO problem as an example to illustrate the working mechanism of $P_\text {HH}$ as shown in Fig. 2. The entire input *G* that could be massive is shown in Fig. 2a. Figure 2b gives an essential subset $\bar{\Gamma }$ that is a naive solution where every node is covered by the arcs related to the source and the sink. Figure 2c is an augmented reduced input in which the augmented region of blue arcs is added by augmentation to the original essential subset. Finally, Fig. 2d shows an improved solution graph obtained by *P* considering the augmented subset in Fig. 2c as input.

### Solution Features

The convergence procedure of most HH-based approaches (and other heuristics, such genetic algorithms) only focuses on objective values. As mentioned earlier, ideally we would like to assess solution quality by a few more criteria, such as the similarity between HH solutions and the benchmark solution. In order to evaluate the effectiveness of alternative objective function designs, four main features are used to make systematic comparisons between solutions derived from *P* and $P_\text {HH}$:

(i) $\Phi _1$: Unified solution quality score (regardless of specific objectives in use);
(ii) $\Phi _2$: Structural similarities;
(iii) $\Phi _3$: Number of different solutions obtained during the iterations of $P_\text {HH}$
(iv) $\Phi _4$: Comparison between optimal objectives produced by *P* and $P_\text {HH}$.

#### Feature $\Phi _1$: Unified Solution Quality Score

Often, it is the objective value that reflects solution qualities and guides the convergence of optimization algorithms, but this objective value is often less directly meaningful or useful to practitioners. On the other hand, actual results and/or contents of optimization criteria found in a solution are important to practitioners since they directly reflect certain operational and business targets and preferences. Thus, we design a *unified* scoring system for reflecting optimization criteria values regardless of which specific objective design is in use. This can be regarded as a way of assessing how good an objective function design is in the view of practitioners. By “unified,” we mean the same scoring system for all candidate alternative objective designs in $\mathcal {F} = \{F_1,F_2,\dots \}$.

This unified scoring system consists of several indicators as sub-features under Layer 1, denoted as $\phi _{11},\phi _{12},\dots$ used to reflect practitioners’ way of assessment. They are often problem-specific, and the details of the indicators we use for the TUSO problem will be reported in Section 4.

**Fig. 3:**
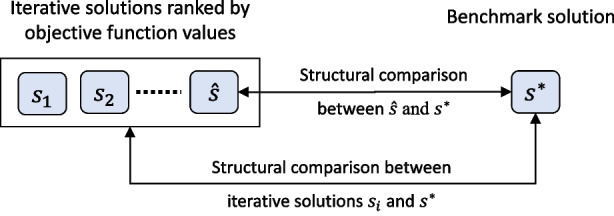
Comparisons of structural similarities

#### Feature $\Phi _2$: Structural Similarities

As we have mentioned in Section 1.1, the benchmark solution $s^*$ found by *P* is the “best” optimal solution we can obtain. $P_\text {HH}$ iteratively solves reduced instances that are updated throughout until convergence. An ineffective objective function often has the tendency of letting $P_\text {HH}$ search over a large “poorly differentiated” solution subspace, resulting in a solution $\hat{s}$ whose structural property is different from $s^*$ even if they have similar or even the same objective value. For instance, the solution of the TUSO problem is based on a subgraph of the original graph. A solution schedule usually contains many unit “diagrams” (daily work plans for train unit vehicles) where how diagrams connect to each other is important because the structural properties of a solution schedule convey the detailed operational plans and these structures are difficult to be fully presented by objective terms. Thus, comparing structural similarities between the heuristic solutions and the exact solution $s^*$ yields the second solution feature to benchmark an objective function’s effectiveness. For an objective function, all the heuristic solutions found by $P_\text {HH}$, i.e., $s_1,s_2,...,\hat{s}$, are compared to the benchmark $s^*$, as illustrated in Fig. 3. The comparison is not made individually on each HH-generated solution $s_i$, but in a way that $s_1,s_2,...,\hat{s}$ are integrated into some metrics to be compared to $s^*$. To achieve this, four indicators served as sub-features in the second layer $\Phi _2$ are devised.

##### Sub-Feature $\phi _{21}$: Overlapping Arc Percentage

The first indicator (sub-feature) $\phi _{21}$ is the overlapping percentage between the final HH-generated solution $\hat{s}$ and exact solution $s^*$. Let $\xi _i$ be the percentage of overlapped arcs in a heuristic solution $s_i$ compared with all the arcs found in $s^*$, i.e., $\xi _i = \dfrac{|A'_i|}{|A^*|}$, where $A^*$ is the set of arcs in the optimal exact solution $s^*$, and $A'_i$ is the set of arcs overlapping with $A^*$ found in a HH solution $s_i$. Then, the first indicator is defined as the largest one of this percentage in a complete HH process among all solutions, i.e., $\xi ^+ = \max _i(\xi _i)$.

##### Sub-Feature $\phi _{22}$: Structural Similarity Range

The second indicator $\phi _{21}$ is the structural similarity range, defined as $r = \xi ^+ - \xi ^-$, where $\xi ^+$ is the same largest percentage aforementioned and $\xi ^-$ is the smallest similarity percentage value, i.e., $\xi ^- = \min _i (\xi _i)$. The range *r* shows how much the similarities are increased through the iterations of $P_\text {HH}$. All alternative objective functions start with the same initial feasible solution such that the lower bounds ($\xi ^-$) of the ranges (corresponding to alternative objective function designs) are comparable.

##### Sub-Feature $\phi _{22}$: Homogeneous Solution Entropy

The third indicator $\phi _{23}$ is the homogeneous solution entropy (or simply “HS entropy”). We define HH-generated solutions (under the same objective function design) having the *same* objective value as *homogeneous solutions* (HS). HS are regarded as being of the “same quality” in terms of their numerical objective values, but they may actually differ in their structures. HS entropy is proposed here specifically for measuring how diverse homogeneous solutions are in a collection of solutions from an objective design. This idea is borrowed from Shannon’s information entropy of a random variable [44], which gives average level of “information,” “surprise,” or “uncertainty” inherent in the variable’s possible outcomes. Given a discrete random variable *X* with possible outcomes $x_1,x_2,...,x_n$ each with the probability $P(x_1 ),P(x_2 ),...,P(x_n)$. The entropy of *X* is defined as $H(X)=-\sum _{i=1}^n P(x_i) \log P(x_i)$. Let *L* be a list of HS with the same objective value, whose members are HS with different structural similarity percentages defined by $\xi _i$ (HS with the same similarity are treated as different members, too). So each *L* corresponds to a unique objective value. Let $\mathcal {L}$ be the set of all such HS lists from a complete HH process. For each $L \in \mathcal {L}$, we define its *homogeneous solution entropy*, denoted by *H*(*L*), to reflect how diverse/uncertain these HS of the same *L* are. To be more specific, let $l \in L$ be a member (i.e., a solution with a similarity percentage) of HS list *L* and $p_l$ be the frequency of *l* among all similarities. The HS entropy for an HS list *L* is defined in the same way as the traditional information entropy,

4 $$\begin{aligned} H(L) = - \sum _{l \in L} p_l \log p_l. \end{aligned}$$

For example, if a HS list $L_1$ has five members (represented by their similarity percentages): $L_1=[50\%, 50\%, 60\%, 70\%, 90\%$], using natural logarithm, its HS entropy is $H(L_1) = -(0.4 \times \ln 0.4 + 3 \times 0.2 \times \ln 0.2) \approx 1.3321$. If another HS list $L_2 = [90\%]$ has only one member, its HS entropy is simply $H(L_2) = -1 \times \ln 1 = 0$.

For an entire HH process with solutions of different objectives values (thus different HS lists $L \in \mathcal {L}$), we use the following weighted average HS entropy as the final metric for measuring the diversity of for this process.

5 $$\begin{aligned} \overline{H} = \frac{1}{|\mathcal {L}|} \sum _{L \in \mathcal {L}} \sigma _L H_L. \end{aligned}$$

In the above, $\sigma _L$ is the standard deviation of the percentage values in list *L* such that a HS list with a higher deviation will be penalized more by having a higher average entropy. For instance, a list $[10\%, 20\%, 50\%, 80\%]$ is less desirable than another list $[70\%, 80\%, 85\%, 88\%]$. This is the final value metric used for sub-feature $\phi _{23}$, and the higher this entropy is, the less desirable. In a “perfect” alternative objective design that is highly efficient in differentiating objective values and structural similarities, or in a simple instance without much complexity, the HS entropy should be close 0. From a less efficient objective design and/or complex instances, it is expected that large positive average HS entropy values will be observed.

##### Sub-Feature $\phi _{34}$: Convergence Rate (“slope”)

**Fig. 4:**
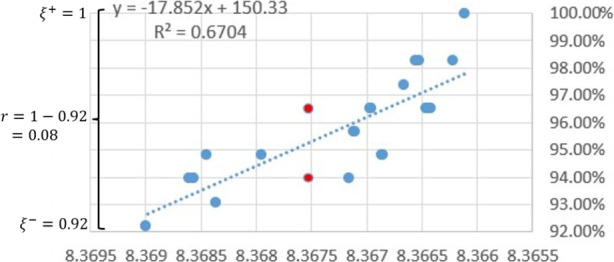
An example of solutions $s_i$ in a complete HH process shown with their objective values $z_i$ (*x*-axis) and percentage similarities $\xi _i$ (*y*-axis). Two homogeneous solutions are highlighted by red

The fourth indicator $\phi _{24}$ is the convergence rate of the entire HH process as reflected by a “slope” of similarity percentage against objective values. To be specific, all the solutions in an HH process are plotted in a graph where the *x*-axis represents the solutions’ objective values and the *y*-axis represents their similarity percentage $\xi _i$. The convergence rate is measured by the linearly fitted line over all the points. Figure 4 gives an example of how the slope is derived. This sub-feature demonstrates how quickly the similarity is improved through the iterative solutions of $P_\text {HH}$.

Some other sub-features under $\Phi _2$ can also be illustrated by Fig. 4. If we take $\hat{\xi } = \xi ^+$, then the value of $\phi _{21}$ is 100%. The range for $\phi _{22}$ is $r = 1-0.92 = 0.08$. There are 17 distinct objective values, giving 17 HS lists. Only the HS list with 8.368 has more than one solutions with different similarity values (96.55% and 93.79%, highlighted in red). The standard deviation of [0.9655, 0.9379] is 0.0138. Therefore, the average entropy of all the HH solutions is $\overline{H} = -\frac{1}{17} \times (16 \times 0 + 0.0138 \times 2 \times 0.5 \times \ln 0.5 = 0.00956$.

#### Feature $\Phi _3$: Number of Different Solutions Obtained During HH Iterations

This feature investigates the capability of a given objective function to differentiate solution properties during HH iterations. Three sub-features are designed to assess the number of different solutions found during an entire HH process. The first sub-feature $\phi _{31}$ is the number of different solutions in terms of objective values. The second sub-feature $\phi _{32}$ is the number of different solutions based on structural similarities. The third one is the average number of HS.

**Fig. 5:**
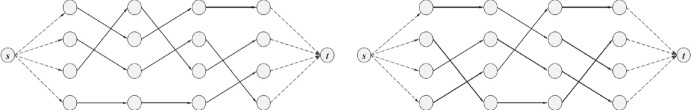
An example of homogeneous solutions

Let us use an example to illustrate HS. Suppose there is a network flow problem where the nodes are customers who need to be served, with a source and sink node added as usual. A directed arc between any two customers means they can be served consecutively. Suppose the optimization target only considers minimizing the number of workers to serve all customers. For this example, the two solutions shown in Fig. 5 both need four workers, but these customers are served in different sequences, thus are regarded as two HS. When connection lengths matter, these two HS could be of very different quality to practitioners. Therefore, apart from the number of workers used, a well-designed objective function for this problem should be able to differentiate as fine-grained as possible the structural properties in the solutions, especially when the solutions are near-optimal.

**Fig. 6:**
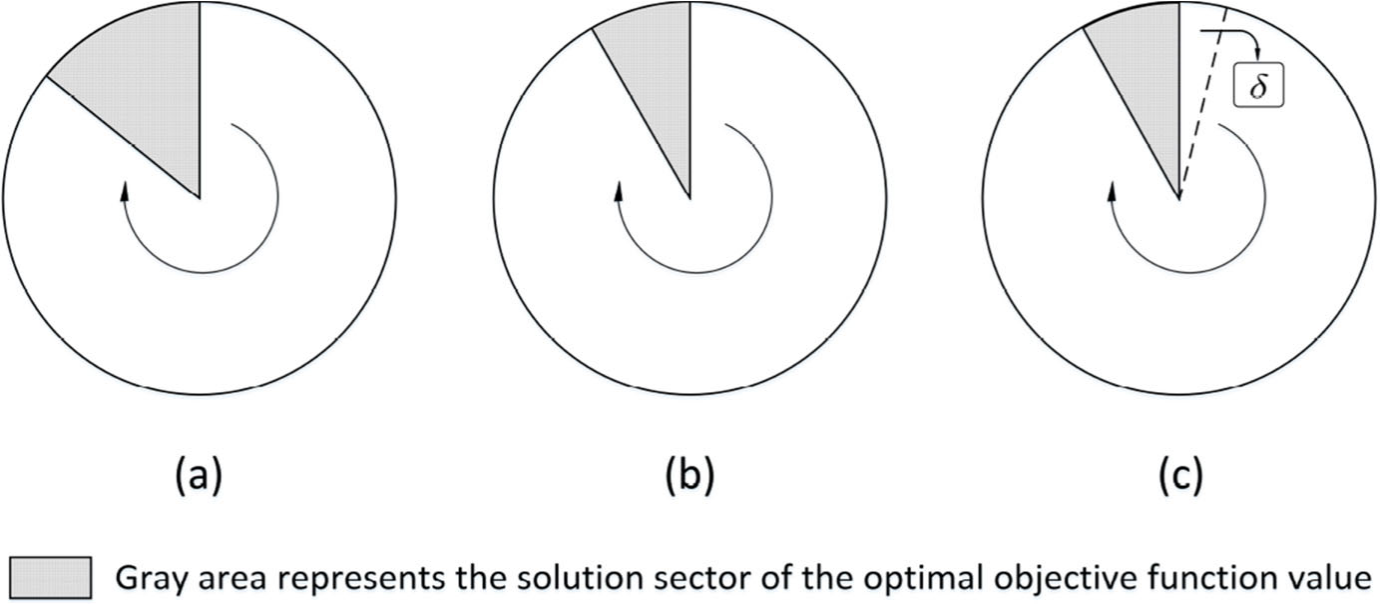
Ranked solution space

Suppose the complete solution space and the optimal solutions are both known. Figure 6a and b show an example of a solution space in which all feasible solutions are ranked by (as indicated by the arrows) two alternative objective functions $F_1$ (a) and $F_2$ (b), respectively.

The gray area represents the solution sector with the theoretically optimal objective function value. The other solutions are getting worse along with the arrow direction. The size of the top-rank solution sector and hence the number of optimal HS of Fig. 6b are smaller than that in Fig. 6a, indicating that the second objective function can differentiate the structural properties better. To find all HS in the top sector, some exhaustive (or brute-force) search methods can be used to explore the entire solution space. However, it is computationally expensive. Usually, a solver will terminate if any solution in the top sector is found, which means the other HS are abandoned without further investigation. Moreover, the real optimality condition (e.g., upper bound (*ub*) = lower bound (*lb*) on a branch-and-bound tree) is hard to achieve in practice for large scale and complex problems. Thus, some less strict stopping criteria are used instead. For instance, the “relative gap” from the optimum, denoted as $\delta$, is often calculated by $\delta = \dfrac{ub - lb}{lb}$, where *ub* and *lb* are upper bound and lower bound, respectively. Because of this, even an “exact” method may return only a sub-optimal solution whose objective function value is worse than that of the solutions in the top sector. “How much worse it could be” depends on the value of $\delta$ (also shown in Fig. 6c). We would like to point out that this optimality gap controlled by $\delta$ commonly set in many exact solvers actually allows a HH method to find some solutions better than $s^*$, the optimal solution found by *P* with a tolerance $\delta$ taking the entire *G* as its input.

In each iteration, $P_\text {HH}$ rotates to a different region of the entire problem space based on an essential subset to form a reduced input. Each reduced input corresponds to a solution subspace. Equivalently, $P_\text {HH}$ extracts many subspaces from the complete solution space. Each solution subspace is searched by *P* to claim one “(near-) optimal” solution as the representative in the top-rank solution sector of this subspace. Thus, $P_\text {HH}$ iteratively ranks the representative solutions from different solution subspaces. For some ineffective objective functions, the representative solutions from different solution subspaces may have the same objective value, although their structures are significantly different. This may lead to quick convergence, but the presence of many HS is not a good sign for objective function effectiveness. In conclusion, for an effective objective function, it is expected to find many different HH solutions both with the same or similar objective values and solution structures. Therefore, the feature on the number of HS for each objective function is included as $\phi _{33}$.

#### Feature $\Phi _4$: Comparison Between Final Objective Values Produced by *P* and $P_\text {HH}$

The objective values of the final solutions from $P_\text {HH}$ are compared with that from *P* for each objective function design. The comparison results have three possible values:

$$\Phi _4=\{ \textrm{better} (\phi _{41}), \textrm{same} (\phi _{42}), \textrm{worse} (\phi _{43}) \}.$$

The reason why it could be “better” is because of some relaxed stopping criteria discussed earlier, for example, the “relative gap” introduced in Fig. 6c with a tolerance $\delta >0$. The comparison results will be converted to a binary array $(v_{41},v_{42},v_{43}) \in \{0,1\}^3$ in one-hot encoding style. For example, if the result is “better,” the array will be (1, 0, 0).

**Fig. 7:**
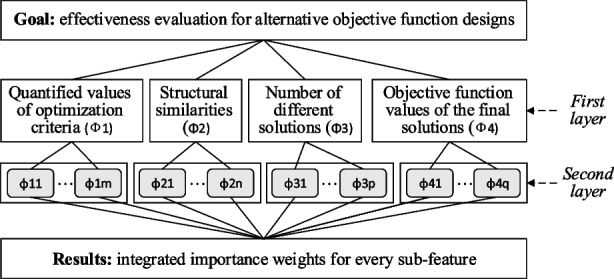
Hierarchical structure of solution features

Finally, Fig. 7 illustrates the hierarchical structure of solution features.

### Evaluation

Each solution feature in $\Phi _1,\dots ,\Phi _4$ reflects the effectiveness of the objective function in certain aspects. Therefore, a scheme of integrating all solution features is proposed to comprehensively benchmark the effectiveness of objective function design. This scheme consists of the following two steps:

#### Step 1: Estimate Feature Relative Importance by AHP

Step 1 estimates the relative importance of all the solution features $\Phi _1,...,\Phi _4$ based on the understanding of real-world problem and algorithm behaviors, where AHP [9] is applied to ensure consistency and accuracy. This process is a widely used systematic approach for quantitatively measuring the relative importance among different factors to support the decision-making for multi-criteria problems. The relative importance between any two same-layer features is measured on the basis of a 1 to 9 scale, where 1 means they are equally important and 9 means one is extremely more important compared to the other. For instance, if feature *i* is *w* $(1\le w\le 9)$ times more important than feature *j*, the relative importance of *i* over *j* is $w_{ij}=w$. Conversely, the relative importance of *j* over *i* is $w_{ji}=1/w$. Hence, a comparison matrix can be obtained, written as a square matrix *W* in Eq. 6, in which $w_{ij}=1/w_{ji}$. To obtain the importance weight vector, each element in *W* is firstly normalized by its column summation to be converted as $W^{nor}$, i.e., $w'_{ij}=w_{ij}/\sum _{i=1}^n w_{ij}$.

6 $$\begin{aligned} W= \begin{bmatrix} 1 &  w_{12} &  \dots &  w_{1n} \\ w_{21} &  1 &  \dots &  w_{2n} \\ \dots &  \dots &  w_{ij} &  \dots \\ w_{n1} &  w_{n2} &  \dots &  1 \end{bmatrix} \longrightarrow \text   W^{nor}= \begin{bmatrix} w'_{11} &  w'_{12} &  \dots &  w'_{1n} \\ w'_{21} &  w'_{22} &  \dots &  w'_{2n} \\ \dots &  \dots &  w'_{ij} &  \dots \\ w'_{n1} &  w'_{n2} &  \dots &  w'_{nn} \end{bmatrix} \end{aligned}$$

The estimated weight vector is calculated by $w_i = \dfrac{\sum _{j=1}^{n} w'_{ij}}{n}$ based on $W^{nor}$. The consistency ratio (*CR*) refers to the reliability of the measured weights through the pairwise comparison method. Generally, the measured weights are acceptable if $CR \le 10\%$ [45]. The features reflecting the effectiveness of objective function have two layers, and each feature at the first layer has some sub-features, as explained in Section 3.3. The global weight of each sub-feature $\hat{w}_{ij}$ is its local weight multiplied by the local weight of its parent feature, $\hat{w}_{ij} = w_i \cdot w_{ij}$.

#### Step 2: Quantify Integrated Effectiveness

Step 2 quantifies the integrated effectiveness for alternative objective function designs based on the derived solution sub-features values $v_{11},v_{12},\dots ,v_{4q}$ corresponding to $\phi _{11},\phi _{12},\dots ,\phi _{4q}$. The magnitudes of solution feature values may differ; thus, they are normalized to ensure comparability. Features are categorized into two sets based on their optimization direction relative to effectiveness:

- **Positive features (PF)**: Features preferred to be *minimized* (smaller values indicate better effectiveness) and thus take a positive sign in the objective function in a minimization problem, e.g., solution entropy.
- **Negative features (NF)**: Features preferred to be *maximized* (larger values indicate better effectiveness) and thus take a negative sign in the objective function in a minimization problem, e.g., structural similarity, solution quality scores.

To unify these directions under a single minimization framework, we apply the equivalence $\max f = \min (-f)$. Thus,

- *PF* features contribute directly to the minimization objective.
- *NF* features are converted via negation to align with minimization: maximizing $v_{ij}^{NF}$ $=$ minimizing $-v_{ij}^{NF}$.

The integrated effectiveness $M_k$ for an objective function $F_k \in \mathcal {F}$ combines both sets as

7 $$\begin{aligned} M_k(\hat{w},v) = \underbrace{\sum _{ij \in PF} \hat{w}_{ij} v_{ij}}_{\text {Minimize directly}} + \underbrace{\sum _{ij \in NF} \hat{w}_{ij} (-v_{ij})}_{\text {Maximize via negation}} = \sum _{ij \in PF} \hat{w}_{ij} v_{ij} - \sum _{ij \in NF} \hat{w}_{ij} v_{ij} \end{aligned}$$

where $\hat{w}_{ij}$ is the AHP-derived weight for $\phi _{ij}$. The design $F^*$ with minimal $M_k$ is optimal:

$$ F^* = \mathop {\textrm{argmin}}\limits _{F_k \in \mathcal {F}} M_k(\hat{w},v). $$

### Scalability Considerations

The proposed methodology relies on solving reduced instances $\overline{G}$ using exact solver *P* within the $P_{\text {HH}}$ framework. While these reduced instances are significantly smaller than the original problem *G*, they remain NP-hard combinatorial optimization problems. This section addresses key scalability considerations and limitations:

#### (a) Computational Tractability Boundariesx

- *Problem-Specific Complexity*: For problems where even small instances exhibit high complexity, reduced instances may still exceed *P*’s solving capacity. The tractability threshold varies by problem class and solver capability.
- *Size-Quality Tradeoff*: The reduction parameter $\mu$ controls $|\overline{G}|/|G|$. Aggressive reduction (e.g., $\mu \ll 0.1$) risks excluding optimal components, while conservative reduction (e.g., $\mu > 0.3$) may yield instances too large for practical solution. This tradeoff requires careful calibration.

#### (b) Mitigation Strategies

- *Approximate Solving*: When *P* cannot solve $\overline{G}$ within time limits, replace it with
  $$\begin{aligned} P'(\overline{G}) = {\left\{ \begin{array}{ll} \text {Heuristic method} &  \text {if } t_{P} > t_{\max } \\ P(\overline{G}) &  \text {otherwise} \end{array}\right. } \end{aligned}$$
  Effectiveness metrics remain valid if benchmark $s^*$ exists, though structural comparisons may be less reliable.
- *Adaptive Reduction*: Dynamically adjust $\mu$ based on *P*’s runtime:
  $$\begin{aligned} \mu ^{(i+1)} = \mu ^{(i)} \times \exp \left( -\beta \times \frac{t^{(i)} - t_{\text {target}}}{t_{\text {target}}}\right) \end{aligned}$$
  where $\beta$ is a sensitivity parameter ($\beta > 0$), and $t_{\text {target}}$ is the desired solution time.
- *Solution Space Pruning*: Restrict $\overline{G}$ using domain-specific rules (e.g., “only connections $\le$ 4 h” in TUSO) to maintain feasibility while reducing size.

#### (c) Applicability Conditions

The methodology is most effective when

(i) Reduced instances $\overline{G}$ are solvable by *P* within $t_{\max }$
(ii) The problem exhibits *component sparsity*: Optimal solutions use $|\Gamma ^*| \ll |G|$ components
(iii) Key structural properties are retained in $\overline{G}$ (e.g., validated by $sim(\hat{s}, s^*) \ge \text { a threshold }\delta _{\min }$)

For problems violating these conditions (e.g., dense constraint satisfaction), alternative evaluation approaches may be needed.

## Computational Experiments

This section reports the computational experiments for investigating the effectiveness of alternative objective function designs using the TUSO problem as the studied case. Before presenting the results, we first briefly introduce the TUSO problem.

### Train Unit Scheduling Optimization

A train unit has a fixed number of passenger carriages, which cannot be split but can be coupled with other train units to make a longer formation. TUSO assigns a limited number of train units to cover all the trips in a timetable satisfying a set of constraints such as fleet size, passenger demand, unit-route compatibility, and turnaround time. The goal is to obtain a set of unit diagrams (referred to as a unit schedule) with minimized operational costs. A unit diagram contains information on serving sequences of trips and some auxiliary activities, for example, coupling/decoupling operations. TUSO is modeled as an integer multi-commodity network flow problem based on a directed acyclic graph (DAG) [2], in which commodities represent different types of train unit. Nodes represent trips, plus a source and a sink by modeling convention. Arcs represent potential connections among the nodes that are generated according to some real-world requirements and constraints. A path on the DAG represents a unit diagram from the source to the sink. See Appendix A for a detailed integer multicommodity flow formulation for the TUSO problem. The alternative objective function designs for the TUSO including different optimization criteria are shown in Table 1.

**Table 1:** Objective function candidates in $\mathcal {F}$

Objectives	Fleet size ($f_1$)	Arc usage ($f_2$)	Mileage ($f_3$)	Connection compactness ($f_4$)
$F_1$	Yes	Yes	No	No
$F_2$	Yes	Yes	Yes	No
$F_3$	Yes	Yes	No	Yes
$F_4$	Yes	Yes	Yes	Yes

There are four candidate criteria to be considered: fleet size ($f_1$), arc usage ($f_2$), total carriage-mileage ($f_3$, mileage for short), and connection compactness ($f_4$). Fleet size is usually the most important optimization criterion because of expensive leasing and maintaining costs. Arc usage gives the structural connectivity relations in the diagrams (and DAG). Both $f_1$ and $f_2$ are core criteria that often cannot be dropped in a reasonably designed objective function. Mileage implies fuel/electricity consumption. The principle of *connection compactness*, which is greatly favored by practitioners, emphasizes two key objectives: first-in-first-out (FIFO) connection sequencing at stations and minimization of excessively long connection waiting times. Globally, this favors solutions with evenly distributed connection durations rather than those exhibiting extreme variations. For instance, for any two connection pairs (*i*, *j*) and (*k*, *l*), solutions minimizing the range of waiting times:

$$ \max (\tau _{ij}, \tau _{kl}) - \min (\tau _{ij}, \tau _{kl}) $$

are preferred, where $\tau _{ij}$ denotes the turnaround/idling time between trips *i* and *j*. For example, connecting trip *i* to *j* in 7 min and trip *k* to *l* in 9 min is preferable to connecting *i* to *j* in 5 min and *k* to *l* in 11 min, despite the identical total turnaround time (16 min). This preference for temporal uniformity constitutes the core compactness criterion. This preference can be generalized to cases with more than two pairs of connection possibilities. See [46] for generalized descriptions in a wider context of integer multicommodity flow problems.

**Table 2:** Details of solution features

*ij*	1st layer features	2nd layer features	PF/NF	$\hat{w}_{ij}$
11	$\Phi _1$ Unified solution quality score	$\phi _{11}$ Fleet size	*PF*	0.2479
12		$\phi _{12}$ Mileage		0.1239
13		$\phi _{13}$ Arc usage		0.0620
14		$\phi _{14}$ Connection compactness		0.1239
21	$\Phi _2$ Structural similarities	$\phi _{21}$ Overlapped arc percentage	*NF*	0.1605
22		$\phi _{22}$ Range		0.0267
23		$\phi _{23}$ Entropy		0.0535
24		$\phi _{24}$ Slope		0.0267
31	$\Phi _3$ Num of different solutions	$\phi _{31}$ In terms of objective function value	*NF*	0.0163
32		$\phi _{32}$ In terms of schedule structure		0.0306
33		$\phi _{33}$ Average # of HS	*PF*	0.0862
41	$\Phi _4$ Comparison between *P* and $P_{HH}$	$\phi _{41}$ Better	*PF*	0.0225
42		$\phi _{42}$ Same		0.0134
43		$\phi _{43}$ Worse		0.0068

### AHP Weighting Process

#### Matrix Development Process

This section addresses the construction and justification of the pairwise comparison matrices used in our AHP. The pairwise comparison matrices for AHP were populated based on our experience from structured engagements with practitioners from TranPennine Express and Greater Anglia (our industry partners) and our algorithmic performance analysis. We have eventually evaluated the relative importance of features through:

(i) Operational criticality assessment: Features were ranked according to their direct impact on day-to-day operations.
(ii) Algorithmic validation: Feature behavior was correlated with convergence patterns observed during the solution of TUSO problem instances.
(iii) Historical schedule analysis: Patterns and structural characteristics (e.g., connection compactness between consecutive trips) were extracted from high-quality historical train unit diagrams.

#### Key Feature Comparison Insights

The final weights presented in Table 2 reflect practitioner-informed priorities derived from our AHP analysis. The following examples illustrate how operational insights and algorithmic observations manifested in specific feature comparisons:

- **Fleet size dominance**: The significant prioritization of fleet size ($\phi _{11}$) at 0.2479–four times higher than arc usage ($\phi _{13}$) at 0.0620–directly reflects practitioners’ operational reality. This substantial weighting difference emerged from quantified assessments showing each reduced train unit translates to a huge amount of leasing and maintenance savings, outweighing arc usage despite their technical importance in solution structure.
- **Connection compactness priority**: The equal weighting of connection compactness ($\phi _{14}$) and mileage ($\phi _{12}$) at 0.1239 each demonstrates a critical operational insight. Though not directly monetized, compact connections were prioritized due to their proven impact on scheduling resilience and overall operational patterns. This is also a preferred feature proposed by practitioners.
- **Structural similarity prioritization**: The overlapped arc percentage ($\phi _{21}$) was assigned a weight six times greater than the range metric ($\phi _{22}$) (0.1605 vs. 0.0267), reflecting the determination that solution completeness in terms of arc overlapping provides a more meaningful measure of structural alignment than solution space coverage.
- **Convergence direction priority**: The 3.3:1 weighting ratio favoring $\phi _{41}$ (“better”) over $\phi _{43}$ (“worse”) reflects a fundamental design philosophy. It emphasizes that a high-quality objective function should actively improve solutions rather than merely avoid degradation.

These examples demonstrate how specific pairwise comparisons (e.g., $\phi _{11}$ vs $\phi _{13}$, $\phi _{21}$ vs $\phi _{22}$) systematically translated domain expertise into quantifiable priorities. The complete set of comparisons maintained logical consistency across all hierarchy levels while accommodating the nuanced trade-offs inherent in real-world scheduling contexts.

#### Validation and Consistency

The solution feature details designed for TUSO are listed in Table 2. TUSO is modeled based on a DAG, and its solution is a sub-graph of the full DAG. The integrated weights of sub-features (column $\hat{w}_i$) and how each sub-feature reflects the objective function effectiveness (column PF/NF) are also given. Using the classical theory of AHP, the consistency ratio (CR) for the first layer features is *CR* = 9.5%, and *CR* values for the second layer features are 0%, 10%, 0.43%, and 1% respectively. They are in the acceptable, range indicating the reliability of the estimated weights.

Complete pairwise comparison matrices, associated eigenvectors, and consistency validation details are provided in Appendix C.

### Experiment Dataset

For a criteria combination $I_k \subset \{1,2,3,4\}$, its objective function is thus $F_k = \sum _{i \in I_k} \alpha _i f_i$. Among the four objectives $f_1, f_2, f_3 \text { and } f_4$, to ensure an appropriate yet not entirely fixed hierarchy, and based on the experience from our numerical analysis and discussions with practitioners, their corresponding weights $\alpha _1,\dots ,\alpha _4$ are designed in the following way in our experiments. Note that strict normalization is not applied as a result of the above analysis and discussions.

- Fleet size ($f_1$): $\alpha _1 = 1$ reflects its dominant role.
- Arc usage ($f_2$): $\alpha _2 = 0.001$ indicates its secondary but essential contribution.
- Mileage ($f_3$): $\alpha _3 = 0.001\times \left( \frac{\sum _{j \in N} m(j)}{|N|} \right) ^{-1}$, where *m*(*j*) is trip *j*’s mileage. This represents the attenuated inverse of average trip mileage.
- Connection compactness ($f_4$): Defined as $f_4 = \sum _{p \in P} \sum _{a \in p} \tau ^2$ (sum of squared turnaround times for all arcs). Letting $S_{\max } = \max _{a \in A} \tau _a$, we set $\alpha _4 = 0.01 / S_{\max }$. This formulation, based on Theorem 1 (Appendix A), promotes compact connections. For instance, for equal sums (e.g., 3+7=10 and 4+6=10), $3^2 + 7^2 = 58 > 4^2 + 6^2 = 52$ favors balanced values.

Although $P_\text {HH}$ is more suitable for large instances, experiments using small instances were conducted so that the exact solver *P* alone was able to yield benchmark solutions. When the best objective function design has been selected, it can be thus applied to large-scale instances with more likelihood to give similar results both in objective values and structures, as if they were given by an exact solver. Thus, three small real-world datasets D1–D3 shown in Table 3 were used in the experiments. These datasets are from a UK train operating company Greater Anglia mainly running in East Anglia and other parts in East England.

**Table 3:** Datasets information

Dataset	No. of trips	DAG arc #	Unit type	Loc#$^1$	Ban loc#$^2$	Unit capacity	Fleet size
D1	109	1080	755/3	11	3	153	7
D2	133	1684	755/4	12	4	205	17
D3	137	1514	360/4	12	6	272	20

$^1$ Number of locations

$^2$ Number of locations banned for coupling/decoupling operations

A train unit scheduling system RS-Opt [2] coded in Mosel based on FICO Xpress-MP 8.5 was used as the exact solver *P*. For the HH framework, the auxiliary hybrid heuristic part $P_\text {HH}$ was derived from SLIM [37] coded in C#. RS-Opt and SLIM were specifically adapted to include the features for the objective function effectiveness evaluation. For instance, to suit multiple alternative objective function designs, well-calibrated weights to maintain the discernible importance of different optimization criteria, connection compactness, and mileage were implemented. The experiments were conducted on a 64-bit workstation PC with 16 G RAM and an Intel Core i7-6700 CPU. RS-Opt has an independent customized branch-and-price component and only utilizes the simplex solver of Xpress-MP to solve LP-relaxation in branch-and-bound and column generation without employing the default integer programming solver provided by Xpress-MP [2]. One of the stopping criteria of RS-Opt was set as the “relative gap” explained in Fig. 6c such that SLIM may converge to solutions whose objective function values are better than the benchmark solution claimed by RS-Opt solely. We set a value of 0.001 for this relative gap to ensure essential quality of the solutions obtained in the experiments. This value was consistently applied throughout our computational study. To get diverse HH solutions, SLIM was set to run 10 times, and each run with a maximum of 3000 reduced RS-Opt iterations. Mixed extraction strategies were used including only selecting the best solutions so far and selecting random solutions. A greedy method [37] is employed to construct the initial feasible solution to start the process to ensure that each candidate objective function has the same start point.

### Solution Feature Results

**Fig. 8:**
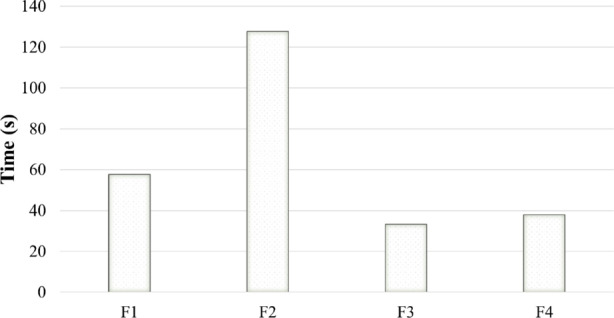
RS-Opt average running time

For SLIM, running time was not considered as an indicator for the objective function effectiveness because a random method was used in *Extraction*() attempting to obtain iterative solutions as many as possible, which may lead SLIM to stop at the maximum iteration. However, running time on RS-Opt is important to benchmark objective function effectiveness because it shows how quickly the alternative objective function designs guide RS-Opt to find $s^*$. The average running times by RS-Opt over the three datasets solved by the four objective function designs are given in Fig. 8. The results show that $F_3$ can quickly guide RS-Opt to find $s^*$, while $F_2$ takes almost 4 times of $F_3$’s running time to converge.

#### Unified Solution Quality Score $\Phi _1$

Under this high level criteria $\Phi _1$, four sub-criteria are considered: fleet size $\phi _{11}$, arc usage $\phi _{12}$, mileage $\phi _{13}$, and connection compactness $\phi _{14}$, reflected by total slack time over all used arcs. Connection compactness is modeled by the time length (slack time) of arcs. At the convergence of both RS-Opt and SLIM for each objective function candidate, the solution quality scores of all the main optimization criteria are derived from their final solutions. In the experiments, all of the four alternative objective function designs $F_1,\dots ,F_4$ would eventually lead RS-Opt and SLIM to converge to solutions having the *same* values in three of the four sub-criteria, $\phi _{11},\phi _{12}$ and $\phi _{13}$, as shown in Table 4.

**Table 4:** Unified solution quality scores of $\phi _{11},\phi _{12}$ and $\phi _{13}$ at convergence of $F_1,\dots ,F_4$

Dataset	Fleet size ($\phi _{11}$)	Mileage ($\phi _{12}$)	Arc usage ($\phi _{13}$)
D1	7	6902.6	116
D2	17	13382.1	190
D3	20	13691.2	230

**Table 5:** Unified solution quality scores on $\phi _{14}$ slack time (in minutes) at convergence of $F_1,...,F_4$

Dataset		D1		D2		D3	
Solver		RS-Opt	SLIM (B/A/W)	RS-Opt	SLIM (B/A/W)	RS-Opt	SLIM (B/A/W)
$\mathcal {F}$	$F_1$	2034	2000	8782	7693/8346/8946	9899	9507/9993/10930$^\dagger$
	$F_2$	2160$^\dagger$	2000	9197	7913/8384/9634$^\dagger$	9597	9872/10015/10501
	$F_3$	1813$^\star$	1843	7180	7046/7193/7309	8897	8905/9020/9099
	$F_4$	1813$^\star$	1813$^\star$/1828/1843	7257	6716$^\star$/6993/7223	9064	8789$^\star$/8993/9133
Gap		347	187/172/157	2017	1197/1391/2411	1002	1083/1022/1831
Max gap		347		2918		2141	

Notes: $\star$/$\dagger$ marks the minimum/maximum slack time for each dataset; OFs: objective functions

The optimization terms corresponding to sub-criteria $\phi _{11}$ and $\phi _{13}$ are both included in all of the four objective designs $F_1,\dots ,F_4$. Therefore, it is reasonable to have no difference because they are supposed to be minimized. We, however, notice that there is no difference in $\phi _{12}$ (mileage) as well no matter whether its corresponding optimization term is or not included into the objective functions. One reason may be that this optimization criterion is positively correlated to some other (sub-)criteria, especially $\phi _{13}$ (arc usage). This phenomenon shows that it is actually possible to minimize mileage without explicitly including its corresponding objective term $f_3$ and to even achieve the same quality as in an objective including an explicit term for that. Note although this example contains only four sub-criteria, in many more complex real-life problems, the number of criteria could be of tens or hundreds. In these cases, such an ability in optimizing a criteria without explicitly including it in the objective will be valuable.

Table 5 shows the quantified values of connection compactness (slack time) for the three datasets. “B/A/W” represents the “best/average/worst” slack time values in the final solutions from multiple runs of SLIM. “Max gap” shows the maximum difference between the maximum and minimum values from RS-Opt and SLIM based on the four alternative objective function designs. The results show that the last sub-criterion $\phi _{14}$, which can be realized by minimizing slack time in the objectives, has significant different values among the solutions from alternative objective function designs, where the biggest “max gap” for D2 is almost 3000 min. Rather different from $\phi _{11},\phi _{12}$ and $\phi _{13}$, the last sub-criterion $\phi _{14}$ (connection compactness) varies significantly among the solutions converged by different objective functions. This indicates that, on the contrary to $\phi _{12}$ (mileage), which might be negligible, the inclusion of the corresponding objective terms of $\phi _{14}$ is necessary if it is to be optimized. Also, this means $\phi _{14}$ is an important indicator for objective function effectiveness.

**Fig. 9:**
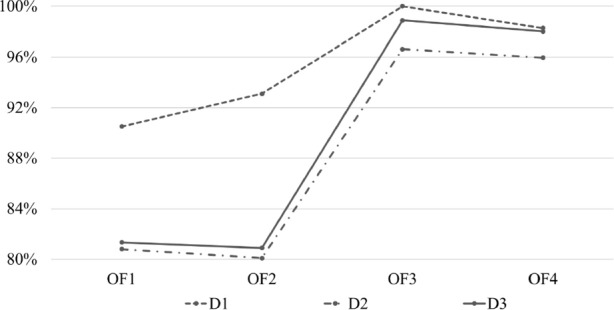
Overlapped arc percentage

**Fig. 10:**
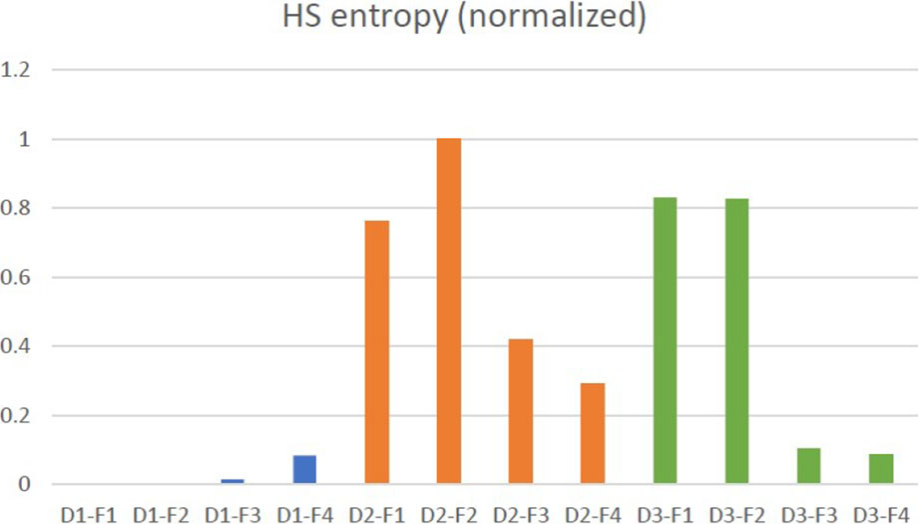
Results on HS entropy values from runs on different objective designs and instances

#### Structural Similarities $\Phi _2$

Structural similarities feature $\Phi _2$ has four sub-criteria: overlapping arc percentage ($\phi _{21}$), similarity range ($\phi _{22}$), HS entropy ($\phi _{23}$), and slope ($\phi _{24}$). Figure 9 gives the results of overlapping arc percentage in the final solutions obtained from all the four objective designs over the three instances. It can be observed that $F_3$ and $F_4$ outperformed $F_1$ and $F_2$ for all the three tested datasets. Note that both $F_3$ and $F_4$ contain the optimization term on connection compactness ($\phi _{14}$) in their objectives, while $F_1$ and $F_2$ do not (see Table 1). This provides another strong evidence that “connection compactness” plays an important role in differentiating structural properties. Considering Figs. 8 and  9 together, $F_3$ always took the shortest time to finish and yielded to the highest similarity percentage, which was unlikely by coincidence. Moreover, Fig. 10 gives the results of HS entropy of the runs over the four objective designs and three instances. It can be observed that the HS entropy values of $F_3$ and $F_4$ tend to be much smaller than the ones of $F_1$ and $F_2$ (except in $D_1$ and $D_2$ where the simple instances only gave unique solution-percentage pairs for $F_1$ and $F_2$ thus giving zero entropy), meaning that $F_3$ and $F_4$ are better in differentiating HS as reflected in their HS entropy.

**Fig. 11:**
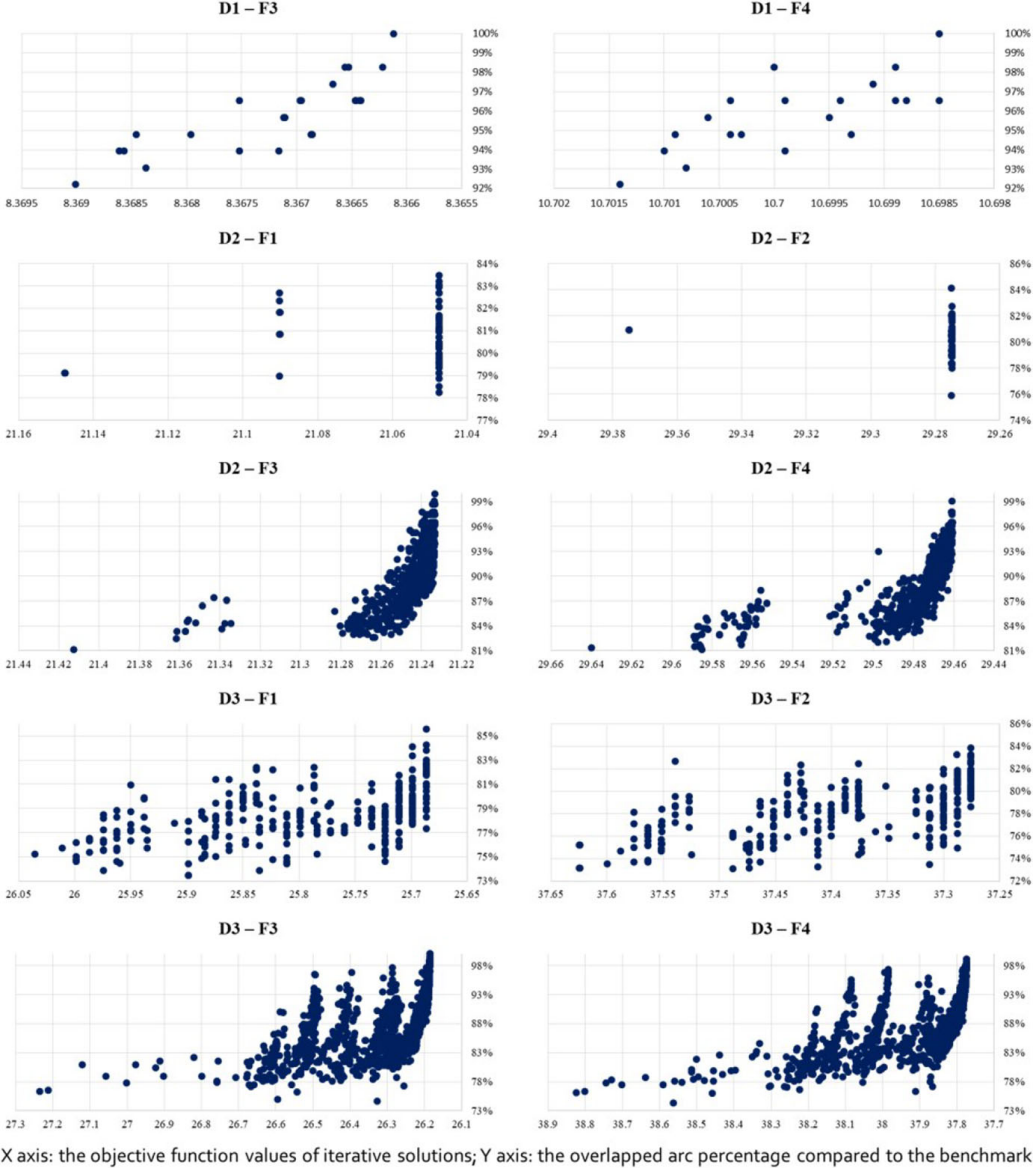
Structural similarities between the iterative solutions and the corresponding benchmark solution

To further have some in-depth look into this phenomenon, Fig. 11 shows the structural similarities between the iterative solutions and the corresponding benchmark solutions for three datasets. The figures of $F_1$ and $F_2$ for D1 are not shown because SLIM only finds one solution whose structural similarity to the corresponding $s^*$ can be found in Fig. 9. Compared to $F_1$ and $F_2$, a few aspects endorsing a better objective function effectiveness are observed for $F_3$ and $F_4$: (i) many more iterative solutions are found; (ii) the solution space is ranked more precisely; (iii) the ranges of structural similarity are larger, which means the similarities have been largely increased via the process of objective function value getting better.

#### The Number of Different Solutions Obtained During the Iterations of $P_\text {HH}$

Table 6 shows the sub-feature values of $\Phi _3$, including the number of solutions in terms of objective function value ($\phi _{31})$, the number of solutions in terms of structural similarity ($\phi _{32}$), and the average number of HS ($\phi _{33}$). $F_1$ and $F_2$ can only find very few solutions in terms of the objective function value through many runs, and even only one solution found for D1. However, for each objective function value, there are many more solutions of different structures. This means many different subspaces derived by SLIM converge to the same objective function value but in different structures, i.e., the given objective function leads SLIM to wander over a large “poorly differentiated” solution space. Consider the results marked in bold in Table 6 as an example.

**Table 6:** Number of iterative solutions

Dataset	D1			D2			D3		
Sub-features	$\phi _{31}$	$\phi _{32}$	$\phi _{33}$	$\phi _{31}$	$\phi _{32}$	$\phi _{33}$	$\phi _{31}$	$\phi _{32}$	$\phi _{33}$
$F_1$	1	1	–	6	58	9.667	40	272	6.8
$F_2$	1	1	–	**4**	**44**	**11**	41	269	6.561
$F_3$	26	27	1.039	279	973	3.488	895	1190	1.298
$F_4$	17	21	1.235	436	918	2.106	814	1069	1.245

$F_2$ found four distinct objective function values; however, these four objective function values correspond to 44 different schedule structures.^1^ This phenomenon confuses SLIM to distinguish better structural properties and converge to a better solution. In average, each objective function value has 11 HS. If the entire solution space is searched, this number would be even larger, which means $F_2$ does not rank the solutions well. $F_1$ and $F_2$ can only lead SLIM to find a single solution whose structure is very different from, but objective function value is identical to the benchmark solution. This is considered as a bad indicator for objective function effectiveness; thus, the values of $\phi _{33}$ for $F_1$ and $F_2$ are empty. On the other hand, $F_3$ and $F_4$ can find a lot more different solutions in terms of objective function value and schedule structure. Besides, the average number of HS for each objective function value is also much smaller. For D1 solutions found by $F_3$, only one objective function value has a HS. The others are all unique (one objective function value corresponds to one detailed schedule), which is desirable. According to the structural comparisons, the effectiveness of $F_3$ and $F_4$ is much superior to $F_1$ and $F_2$.

#### Objective Function Values of the Final Solutions

Table 7 gives the results of the objective function values of final solutions from SLIM compared to that from RS-Opt for each dataset over four alternative objective function designs. The comparison results of $F_1$ and $F_2$ have three cases: better, same, and worse.

However, $F_3$ and $F_4$ can always lead SLIM to find solutions whose objective function values are better than their corresponding benchmark solutions. For $F_3$ and $F_4$, the solutions with the same objective function value of $s^*$ are also found by SLIM, and their schedule structures are the same to the corresponding benchmark structures. The feature values for the comparison of objective function values are converted as a matrix with binary values to calculate the integrated objective function effectiveness, as shown in the last three columns of Table 8.

**Table 7:** Final objective function value comparisons of three datasets

	$F_1$	$F_2$	$F_3$	$F_4$
D1	Same	Same	Better	Better
D2	Better	Better	Better	Better
D3	Worse	Worse	Better	Better

### Integrated Effectiveness

Through the analysis of solution features, generally $F_3$ and $F_4$ have better effectiveness than $F_1$ and $F_2$. This section evaluates the integrated effectiveness for alternative objective function designs by employing the method proposed in Section 3.4. To compute the integrated effectiveness according to Eq 7, the weights and normalized values of all the features are needed, in which weights can be found in Table 2.

**Table 8:** Normalized values of sub-features

Data	$\mathcal {F}$	$v_{11}$	$v_{12}$	$v_{13}$	$v_{14}$	$v_{21}$	$v_{22}$	$v_{23}$	$v_{24}$	$v_{31}$	$v_{32}$	$v_{33}$	$v_{41}$	$v_{42}$	$v_{43}$
D1	$F_1$	1	1	1	.942	.905	0	0	0	.039	.037	.8	0	1	0
	$F_2$	1	1	1	1	.931	0	0	0	.0385	.037	.8	0	1	0
	$F_3$	1	1	1	.84	1	1	.178	1	1	1	.841	1	0	0
	$F_4$	1	1	1	.84	1	1	1	.868	.654	.778	1	1	0	0
D2	$F_1$	1	1	1	.955	.808	.277	.763	0	.0138	.06	.879	1	0	0
	$F_2$	1	1	1	1	.801	.435	1	0	.009	.045	1	1	0	0
	$F_3$	1	1	1	.781	1	1	.420	1	.64	1	0.317	1	0	0
	$F_4$	1	1	1	.789	.99	.949	.290	.0236	1	.943	.191	1	0	0
D3	$F_1$	1	1	1	.905	.847	.478	1	.483	.0447	.229	1	0	0	1
	$F_2$	1	1	1	1	.831	.426	.994	.622	.046	.226	.965	0	0	1
	$F_3$	1	1	1	.848	1	1	.123	1	1	1	.191	1	0	0
	$F_4$	1	1	1	.863	.991	.98	.102	.928	.909	.898	.183	1	0	0

To obtain comparable sub-feature values $v_{ij}$, a quantified value of each sub-feature is normalized by the largest value across the four candidate objective functions, as shown in Table 8. The first three columns are the normalized values of fleet size, mileage, and arc usage. Referring back to Table 4, their quantified values across the four candidate objective functions are the same. Thus, they equally reflect the effectiveness of each candidate, i.e., the effectiveness is mainly determined by the other features. The last three columns are the matrix converted from the comparisons of the objective function values of the final solutions from SLIM and RS-Opt, in which each element is binary. For instance, the matrix for D1 means that $F_1$ and $F_2$ guided SLIM to converge at a solution with the same objective function value of benchmark solution, $F_3$ and $F_4$ converged to a solution with a better objective function values than the benchmark solution.

Table 9 eventually demonstrates the results of integrated effectiveness. It shows that the effectiveness of $F_3$ and $F_4$ has consistently received higher overall score than $F_1$ and $F_2$, which well reflects the analysis of the quantified values of solution features. The effectiveness of $F_3$ is slightly better than $F_4$, and similar phenomena is observed between $F_1$ and $F_2$. This indicates that “mileage” ($f_3$) does not significantly contribute to the objective function effectiveness and may even have some negative influence.

**Table 9:** Integrated effectiveness (bold: best values; italic: worst values)

	$F_1 (f_1,f_2)$	$F_2 (f_1,f_2,f_3)$	$F_3 (f_1,f_2,f_4)$	$F_4 (f_1,f_2,f_3,f_4)$
D1	0.4608	*0.4638*	0.3174	**0**.**3031**
D2	0.4254	*0.4262*	**0**.**2579**	0.2780
D3	0.4024	*0.4118*	**0**.**2653**	0.2762
Average	0.4295	*0.4339*	**0**.**2802**	0.2858
Final ranking	$F_3 \succ F_4 \succ F_1 \succ F_2$			

On the other hand, there is great evidence showing that “connection compactness” ($f_4$) plays a key role in helping SLIM differentiate better structural properties to the solutions and further boosts objective function effectiveness. Moreover, these three datasets have consistent effectiveness rankings for the four alternative objective function designs tested, which is $F_3 \succ F_4 \succ F_1 \succ F_2$. This is also consistent with the effectiveness ranked by the average running times of RS-Opt shown in Fig. 8. According to the feedback from the practitioners, $F_3$ is the most effective objective function using only three main optimization criteria to deliver solutions that are considered as good quality even when they consider some other hidden criteria to judge. And the solutions found by $F_3$ and $F_4$ are significantly better than that found by $F_1$ and $F_2$ in practice. This feedback endorses that the proposed methodology can effectively and systematically establish confidence in alternative objective function designs. Through this investigation and discussions with the practitioners, it is concluded that considering fleet size, arc usage, and connection compactness in the objective function is the most effective combination of main optimization criteria. Through the comparison between the alternative objective function designs containing different optimization criteria combinations, we eventually identify that the inclusion of mileage will slightly reduce objective function effectiveness, while connection compactness significantly increases objective function effectiveness.

### Connection to Objective Function Landscapes

The concept of an *objective function landscape* provides a powerful theoretical lens for interpreting our methodology’s effectiveness evaluation. In combinatorial optimization, the objective function defines a multidimensional landscape where solutions correspond to spatial coordinates and their objective values represent elevation [47]. Characterized by features like peaks, valleys, plateaus, and basins of attraction (regions where local search dynamics converge to particular optima), this landscape structure profoundly influences algorithmic behavior and solution quality [48, 49]. Our methodology connects to this concept through key relationships bridging empirical observations with landscape theory.

#### Landscape Transformation via Weighted-Sum Scalarization

Weighted-sum objectives $F_k = \sum _{i \in I_k} \alpha _i f_i$ fundamentally reshape optimization landscapes through three primary mechanisms. First, poorly designed objectives create extensive plateaus–flat regions where structurally distinct solutions share identical objective values: e.g., manifested as high homogeneous solution entropy (Eq. 4). This phenomenon explains the “poorly differentiated solution space” we observed with $F_1$/$F_2$ in TUSO. Conversely, effective weight combinations create landscapes with strong gradients that steer search toward desirable regions, as seen when including connection compactness ($f_4$) in $F_3$/$F_4$. Each weight combination $\{\alpha _i\}$ also represents a scalarization of the underlying multi-objective landscape, where our benchmarking reveals which projections best preserve structural preferences.

#### Landscape Navigation by Hybrid Heuristics

The $P_{HH}$ algorithm’s trajectory through solution space is governed by landscape topography. During backbone extraction ($\bar{\Gamma }$), the method may operate within basins of attraction. Effective objectives create basins retaining structural properties of $s^*$, measured by overlapped arc percentage ($\phi _{21}$). Wheel rotation enables escape from flat regions, with high-differentiation landscapes permitting discovery of structurally distinct solutions ($\phi _{31}$, $\phi _{32}$). The convergence slope ($\phi _{24}$) quantifies descent efficiency—directly determined by landscape gradient strength and ruggedness.

#### Landscape-Aware Effectiveness Metrics

Our feature design implicitly characterizes fundamental properties of objective function landscapes through a few principal metrics that serve as proxies for critical topological features. For instance, structural similarity measures the degree of basin alignment between the heuristic’s trajectory and the benchmark optimum’s basin of attraction. This metric quantifies the congruence between the solution space region explored by the heuristic and the basin containing the benchmark solution, where higher values indicate that the hybrid heuristic consistently navigates within the same topological neighborhood as the benchmark. Homogeneous solution entropy quantifies the extent of solution space plateaus by measuring the information-theoretic diversity of solutions sharing identical objective values. This metric captures the landscape’s neutrality—the degree to which structurally distinct solutions are indistinguishable under the objective function. High entropy values signal extensive flat regions where the objective function fails to provide directional gradients, resulting in search stagnation and reduced solution differentiation capability.

#### Pareto Frontier Heterogeneity and Objective Design

In multi-objective optimization, the Pareto frontier represents solution sets where no objective improves without worsening another [10]. Our framework could reveal certain frontier heterogeneity: near boundary points such as the region that extremely prefers fleet size, landscapes may exhibit steeper gradients but narrower basins, favoring sparse designs like $F_3$. In transitional zones, comprehensive designs like $F_4$ mitigate plateaus through finer differentiation. Structural clustering enables $\phi _{21}$ to identify optimal $I_k$ combinations for preserving region-specific features, suggesting adaptive objective functions that dynamically adjust composition during optimization.

This landscape perspective strengthens our foundation by explaining performance differences (e.g., $F_3$’s advantage through favorable gradients), linking metrics to landscape analysis techniques, and providing predictive frameworks for new criteria.

While these connections establish valuable conceptual grounding, detailed theoretical analysis remains beyond our current scope. We defer rigorous investigation to future work. This connection positions our methodology at the intersection of empirical evaluation and landscape theory—offering both academics and practitioners actionable insights with theoretical guarantees.

## Conclusion and Future Work

For complex real-world scheduling problems that are virtually all NP-hard and each has numerous possible structural properties, it is not easy to establish confidence in the effectiveness of objective function designs. Research on this topic is scarce in the literature. There is a lot of research studying real-world multi-criteria optimization and automatic methods for algorithm parameter control, seen in the literature review in Section 2. However, they mostly address the issue of promoting performance of algorithms to deliver better quality solutions or shorten computational time. In this research, a methodology evaluating objective function effectiveness through comparisons between solutions obtained from an exact ILP solver and an auxiliary hybrid heuristic is presented, where different combinations of main optimization criteria are considered. A set of solution features reflecting objective function effectiveness is designed, where the key metrics in measuring effectiveness are derived from structural comparisons between heuristic solutions and the exact solution. A hierarchical scheme of integrating all features together is devised to quantify the objective function effectiveness. The experiments carried out with TUSO instances strongly support this methodology. The experiments have shown that the main optimization criteria combination in the objective function can be optimized, i.e., it is not necessary to include all these criteria in the objective function to obtain a “good” solution. The review and feedback from the practitioners also endorse this methodology.

While our methodology demonstrates good performance on the TUSO problem, we acknowledge fundamental scalability constraints inherent to NP-hard combinatorial optimization. The core limitation stems from the fact that reduced instances $\overline{G}$, though significantly smaller than the original problem *G*, remain instances of the same NP-hard class. For problems where even modestly sized instances are computationally challenging (e.g., high-dimensional vehicle routing or protein folding), the requirement for repeated exact solving of $\overline{G}$ within $P_{\text {HH}}$ may become impractical.

This limitation manifests primarily in two scenarios: First, for problems exhibiting dense solution spaces where optimal components cannot be isolated through reduction (i.e., $|\Gamma ^*| \approx |G|$), the necessary size of $\overline{G}$ may approach that of *G*. Second, constraint-dense problems often require large $\overline{G}$ to maintain feasibility, potentially exceeding solver capacity. In such cases, the hybrid heuristic may fail to generate meaningful solution trajectories.

To extend the methodology’s applicability, we propose two adaptive strategies: (1) replacing the exact solver *P* with approximation algorithms when solving $\overline{G}$ exceeds time thresholds, and (2) implementing dynamic reduction control that adjusts $\mu$ based on real-time solver performance.

In ongoing work, we are going to investigate methods to improve the auxiliary heuristics to perform better in deriving reduced inputs. Another direction is how to evaluate the objective function effectiveness if no practical exact method solver is available. We may consider a dynamic benchmark that can be updated once a better solution is found until no improvement can be achieved. Finally, we will conduct algorithmic design and experiments regarding the above-mentioned scalability issues.

## Data Availability

Part of data involved in the research is commercially sensitive. Where possible, the data that can be made publicly available is deposited in http://archive.researchdata.leeds.ac.uk/.

## Appendix A. Proof of Method for Encouraging Arc Compactness

### Theorem 1

Let $\textbf{x} = (x_1, \dots , x_n)$ and $\textbf{y} = (y_1, \dots , y_n)$ be vectors in $\mathbb {R}^n$ such that $\sum _{i=1}^n x_i = \sum _{i=1}^n y_i = S$. If $\textbf{y}$ is obtained from $\textbf{x}$ through a finite sequence of disparity-reducing operations, then $\sum _{i=1}^n y_i^2 < \sum _{i=1}^n x_i^2$.

### Proof

It suffices to prove that a single disparity-reducing operation strictly decreases the sum of squares. Consider a disparity-reducing operation applied to components $x_i$ and $x_j$ where $x_i > x_j$. Let $t \in (0, \frac{x_i - x_j}{2}]$ and define

$$ x_i' = x_i - t, \quad x_j' = x_j + t, $$

while $x_k' = x_k$ for all $k \ne i,j$. The change in the sum of squares is

$$\begin{aligned} \Delta&= \left[ (x_i - t)^2 + (x_j + t)^2\right] - \left[ x_i^2 + x_j^2\right] \\&= \left[ x_i^2 - 2x_i t + t^2 + x_j^2 + 2x_j t + t^2\right] - x_i^2 - x_j^2 \\&= 2t^2 + 2t(x_j - x_i). \end{aligned}$$

Let $\delta = x_i - x_j > 0$. Substituting $x_j - x_i = -\delta$ yields

$$ \Delta = 2t(t - \delta ). $$

Since $0 < t \le \delta /2$, we have $t - \delta \le -\delta /2 < 0$. As $t > 0$, it follows that

$$ 2t(t - \delta ) < 0. $$

Thus, $\Delta < 0$, meaning the sum of squares strictly decreases. Since $\textbf{y}$ is obtained by finitely many such operations, $\sum _{i=1}^n y_i^2 < \sum _{i=1}^n x_i^2$. $\square$

## Appendix B. Integer Multicommodity Flow Formulation for the Train Unit Scheduling Optimization Problem

The TUSO problem is formulated as an integer multi-commodity flow problem based on a directed acyclic graph $\mathcal {G}=(\mathcal {N},\mathcal {A})$. The commodities are train unit types denoted by $k \in K$. $\mathcal {N}=N\cup \{s,t\}$ represents the nodes where *s*, *t* are the source and sink with a number of $b^k$ train units to be routed from *s* to *t* for each type $k \in K$. *N* is the set of trip nodes *j* each with a passenger demand $r_j$ and a coupling upper bound $u_j$. $\mathcal {A}=A \cup A_0$ is the arc set where $A=\{(i,j):i,j \in N\}$ contains turnaround connection arcs and $A_0=\{(s,j):j \in N \} \cup \{(j,t): j \in N \}$ contains sign-on/-off arcs. A path is defined restrictively as an *s*-*t* path from the source to the sink, which contains a potential plan of daily work for a train unit. Let $P^k$ be the set of paths associated with type *k* (i.e., the paths can be used to route unit type *k*) and $x_p$ be the flow amount in $p \in P^k$. An integer multi-commodity flow model based on path variables can be formulated as

B1 $$\begin{aligned} \quad \min \quad \hspace{-40pt}  &   \sum _{k \in K}\sum _{p \in P^k}c_p x_p  &    &\end{aligned}$$

B2 $$\begin{aligned} \quad \text {s. t.} \quad \hspace{-40pt}  &   \sum _{p\in P^k}x_p&\le b_k, \quad  &   \forall k \in K; \end{aligned}$$

B3 $$\begin{aligned} \hspace{-40pt}  &   \sum _{k \in K_j} \sum _{p \in P_j^k} q_k x_p&\ge r_j, \quad \quad  &   \forall j\in N; \end{aligned}$$

B4 $$\begin{aligned} \hspace{-40pt}  &   \sum _{k \in K_j} \sum _{p \in P_j^k} v_k x_p&\le u_j, \quad \quad  &   \forall j\in N; \end{aligned}$$

B5 $$\begin{aligned}  &   \text {Coupled units for { j} are compatible},&\quad  &   \forall j\in N; \end{aligned}$$

B6 $$\begin{aligned}  &   \text {No coupling/decoupling at banned locations for { j}},&\quad  &   \forall j\in N; \end{aligned}$$

B7 $$\begin{aligned}  &   x_p&\in \mathbb {Z}_+, \hspace{-185pt}  &   \forall p \in P^k,\forall k \in K. \end{aligned}$$

In Objective Eq. B1, $c_p$ is the cost of one unit of flow on path *p*. This cost is a weighted sum of four optimization criteria:

$$\begin{aligned} c_p =&\underbrace{\alpha _1 \cdot 1}_{\text {Fleet size weight}} + \underbrace{\alpha _2 \cdot |p|_A}_{\text {Arc usage}} + \underbrace{\alpha _3 \cdot d_p}_{\text {Mileage}} + \underbrace{\alpha _4 \cdot \sum _{a \in p} \tau _a^2}_{\text {Connection compactness}} \end{aligned}$$

where

- **Fleet size **($f_1$): Represented by $\alpha _1$ per path. Total fleet is $\sum _k \sum _p x_p$.
- **Arc usage **($f_2$): $|p|_A$ = number of arcs in path *p*. Total arc usage is $\sum _k \sum _p x_p |p|_A$.
- **Mileage **($f_3$): $d_p$ = distance (mileage) of path *p* (sum of trip distances + empty movement distances).
- **Connection compactness **($f_4$): $\tau _a$ = turnaround time on connection arc *a*. Total squared turnaround time is $\sum _k \sum _p x_p \sum _{a \in p} \tau ^2$. See Section 4.1 and Appendix A for a justification of this technique.

Constraints Eq. B2 require the number of units used to be no more than the fleet limit $b_k$ for each type *k*. Constraints Eq. B3 ask the capacity provision ($q_k$ being the unit capacity in number of seats of type *k*) for each trip to be no less than the passenger demand $r_j$. Constraints Eq. B4 limit the number of coupled cars at *j* to be no more the an upper bound $u_j$, where $v_k$ is the number of cars in unit type *k*. Constraints Eqs. B5 and B6 are additional requirements on unit type compatibility and banning coupling/decoupling operations at certain locations. They are satisfied by customized branching strategies rather than explicit linear constraints [2]. Finally, Constraints Eq. B7 give the variable domain.

## Appendix C. AHP Calculation Matrices

### C.1 First-Level Criteria Matrix

**Table 10:** First-level pairwise comparison matrix (CR = 0.095)

	$\Phi _1$	$\Phi _3$	$\Phi _2$	$\Phi _4$	Eigenvector
$\Phi _1$	1	5	3	9	0.558
$\Phi _3$	1/5	1	1/3	5	0.133
$\Phi _2$	1/3	3	1	7	0.268
$\Phi _4$	1/9	1/5	1/7	1	0.042

#### Consistency Validation

$$\begin{aligned} \lambda _{\max }&= 4.255 \\ CI&= \frac{4.255 - 4}{3} = 0.085 \\ CR&= \frac{0.085}{0.90} = 0.095 < 0.1 \end{aligned}$$

### C.2 s-Level Matrices

#### C.2.1 Target Value ($\Phi _1$) Sub-Criteria

**Table 11:** Target value sub-criteria matrix (CR = 0.00)

	$\phi _{11}$	$\phi _{12}$	$\phi _{13}$	$\phi _{14}$	Eigenvector
$\phi _{11}$	1	2	4	2	0.444
$\phi _{12}$	1/2	1	2	1	0.222
$\phi _{13}$	1/4	1/2	1	1/2	0.111
$\phi _{14}$	1/2	1	2	1	0.222

##### Justification

Practitioners prioritized fleet size due to leasing costs, while connection compactness and mileage were equally weighted for operational stability. See Table 11 for details.

#### C.2.2. Structural Similarities ($\Phi _2$) Sub-Criteria

**Table 12:** Structural similarities sub-criteria matrix (CR = 0.10)

	$\phi _{21}$	$\phi _{22}$	$\phi _{23}$	$\phi _{24}$	Eigenvector
$\phi _{21}$	1	6	3	6	0.524
$\phi _{22}$	1/6	1	1/2	1	0.199
$\phi _{23}$	1/3	2	1	2	0.093
$\phi _{24}$	1/6	1	1/2	1	0.093

##### Justification

Overlapped arc percentage deemed most critical for solution completeness, while entropy received higher weight than range/slope for its correlation with diversity-based solution quality evaluations. See Table 12 for details.

#### C.2.3 Solutions ($\Phi _3$) Sub-Criteria

**Table 13:** Solutions sub-criteria matrix (CR = 0.043)

	$\phi _{31}$	$\phi _{32}$	$\phi _{33}$	Eigenvector
$\phi _{31}$	1	1/2	1/5	0.125
$\phi _{32}$	2	1	1/3	0.222
$\phi _{33}$	5	3	1	0.651

##### Justification

Overlapped arc percentage deemed most critical for solution completeness, while entropy received higher weight than range/slope for its correlation with diversity-based solution quality evaluations. See Table 13 for details.

#### C.2.4 Convergence ($\Phi _4$) Sub-Criteria

**Table 14:** Convergence sub-criteria matrix (CR = 0.01)

	$\phi _{41}$	$\phi _{42}$	$\phi _{43}$	Priority
$\phi _{41}$	1	2	3	0.545
$\phi _{42}$	1/2	1	2	0.286
$\phi _{43}$	1/3	1/2	1	0.168

##### Justification

: “Better” outcomes prioritized for solution quality improvement, with “same” rated higher than “worse” for stability. See Table 14 for details.

### C.3 Global Weight Calculation Example

For $\phi _{11}$ (Fleet Size):

$$\begin{aligned} \text {Local weight}&= 0.444 \\ \text {Parent weight}&= 0.558 \\ \text {Global weight}&= 0.444 \times 0.558 = 0.2479 \end{aligned}$$

All CR values were maintained to be not larger than 0.1, confirming judgment consistency throughout the hierarchy.

## Footnotes

1 Note: in Fig. 11 of “D2-F2,” it seems that only “two” objective function values are found. This is because some values are very close. The “four” values are *29.2749, 29.2750, 29.3749, and 29.3750*, respectively. The same reason applies to other countable values in Fig. 11, which looks like not consistent with Table 6.

## Code Availability

Not applicable due to confidentiality reasons.
